# Proteome profiling of enzalutamide‐resistant cell lines and serum analysis identified ALCAM as marker of resistance in castration‐resistant prostate cancer

**DOI:** 10.1002/ijc.34159

**Published:** 2022-06-21

**Authors:** Anita Csizmarik, Dávid Keresztes, Nikolett Nagy, Thilo Bracht, Barbara Sitek, Kathrin Witzke, Martin Puhr, Ilona Tornyi, József Lázár, László Takács, Gero Kramer, Sabina Sevcenco, Agnieszka Maj‐Hes, Zsolt Jurányi, Boris Hadaschik, Péter Nyirády, Tibor Szarvas

**Affiliations:** ^1^ Department of Urology Semmelweis University Budapest, Hungary; ^2^ Medizinisches Proteom Center Ruhr University Bochum Bochum Germany; ^3^ Department of Anesthesia, Intensive Care Medicine and Pain Therapy University Hospital Knappschaftskrankenhaus Bochum Bochum Germany; ^4^ Center for Protein Diagnostics, Medical Proteome Analysis Ruhr‐University Bochum Bochum Germany; ^5^ Department of Urology Medical University of Innsbruck Innsbruck Austria; ^6^ Department of Human Genetics University of Debrecen Debrecen Hungary; ^7^ Biosystems International Kft Debrecen Hungary; ^8^ Department of Urology Medical University of Vienna Vienna Austria; ^9^ Department of Radiobiology and Diagnostic Onco‐Cytogenetics, Center of Radiotherapy National Institute of Oncology Budapest Hungary; ^10^ Department of Urology University Hospital Essen, University of Duisburg‐Essen Essen Germany

**Keywords:** ALCAM, biomarker, enzalutamide, prostate cancer, proteome analysis

## Abstract

Enzalutamide (ENZA) is a frequently used therapy in metastatic castration‐resistant prostate cancer (mCRPC). Baseline or acquired resistance to ENZA have been observed, but the molecular mechanisms of resistance are poorly understood. We aimed to identify proteins involved in ENZA resistance and to find therapy‐predictive serum markers. We performed comparative proteome analyses on ENZA‐sensitive parental (LAPC4, DuCaP) and ‐resistant prostate cancer cell lines (LAPC4‐ENZA, DuCaP‐ENZA) using liquid chromatography tandem mass spectrometry (LC‐MS/MS). The top four most promising candidate markers were selected using bioinformatic approaches. Serum concentrations of selected markers (ALCAM, AGR2, NDRG1, IDH1) were measured in pretreatment samples of 72 ENZA‐treated mCRPC patients using ELISA. In addition, ALCAM serum levels were measured in 101 Abiraterone (ABI) and 100 Docetaxel (DOC)‐treated mCRPC patients' baseline samples. Results were correlated with clinical and follow‐up data. The functional role of ALCAM in ENZA resistance was assessed in vitro using siRNA. Our proteome analyses revealed 731 significantly differentially abundant proteins between ENZA‐sensitive and ‐resistant cells and our filtering methods identified four biomarker candidates. Serum analyses of these proteins revealed only ALCAM to be associated with poor patient survival. Furthermore, higher baseline ALCAM levels were associated with poor survival in ABI‐ but not in DOC‐treated patients. In LAPC4‐ENZA resistant cells, ALCAM silencing by siRNA knockdown resulted in significantly enhanced ENZA sensitivity. Our analyses revealed that ALCAM serum levels may help to identify ENZA‐ and ABI‐resistant patients and may thereby help to optimize future clinical decision‐making. Our functional analyses suggest the possible involvement of ALCAM in ENZA resistance.

## What's new?

Over the years, many new treatments have come on the market for metastatic castration‐resistant prostate cancer, including enzalutamide, an androgen receptor inhibitor with multiple mechanisms of action. Still, patients often develop resistance. Here, the authors used proteome analysis to identify proteins that contribute to enzalutamide resistance. By comparing protein expression in cells sensitive or resistant to enzalutamide, they created a list of 731 proteins of interest, which they later narrowed down to four potential biomarkers. Only ALCAM was associated with poor patient survival. Silencing ALCAM expression, they found, increased enzalutamide sensitivity.

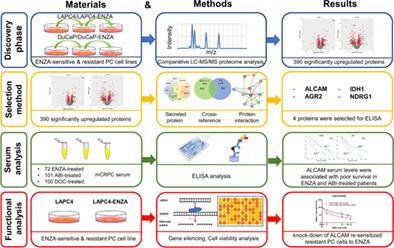

**Abbreviations**
ABI: abiraterone
AGR2: anterior gradient 2
ALCAM: activated leukocyte cell adhesion molecule
AR: androgen receptor
ATM: ataxia‐telangiectasia
BRCA1,2: breast cancer type 1,2
CRPC: castration‐resistant prostate cancer
DHT: dihydrotestosterone
DMSO: dimethylsulfoxide
DOC: docetaxel
ECOG: Eastern Cooperative Oncology Group
ELISA: enzyme linked immunosorbent assay
ENZA: enzalutamide
GO: gene ontology
IDH1: isocitrate dehydrogenase 1
KDM1B: lysine demethylase 1B
KEGG: Kyoto Encyclopedia of Genes and Genomes
LC‐MS/MS: liquid chromatography tandem mass spectrometry
LN: lymph node
MMP‐7: matrix metalloproteinase 7
MMR: mismatch repair
NDRG1: N‐myc downstream‐regulated gene 1
NEPC: neuroendocrine prostate cancer
NFMD/TBST: nonfat dry milk/Tris‐buffered saline with 0.1% Tween 20 detergent
OS: overall survival
PC: prostate cancer
PCWG: prostate cancer working group
RAD: radiation
ROC: receiver operator characteristic
RPE: radical prostatectomy
TCGA: the cancer genome atlas

## 1 INTRODUCTION

In the last decade, the management of metastatic castration‐resistant prostate cancer (mCRPC) has significantly changed. Beside docetaxel (DOC) therapy, novel treatments became available for these patients including cabazitaxel,^1^ next‐generation androgen receptor (AR) inhibitors (abiraterone (ABI),^2^, ^3^ enzalutamide^4^, ^5^); immunotherapy (sipuleucel‐T)^6^; PARP‐inhibitors (olaparib, rucaparib^7^, ^8^), radiopharmaceuticals (radium‐223)^9^ and radioligand therapy.^10^ Despite the obvious benefits achieved with these therapies, mCRPC remains a lethal disease with a poor clinical outcome.

Enzalutamide (ENZA) is a second‐generation androgen receptor inhibitor, which has multiple effects on androgen signaling, including inhibition of ligand binding to AR, blockade of AR nuclear translocation and prevention of AR binding to DNA. In phase III studies, ENZA treatment significantly prolonged overall and progression‐free survival of mCRPC patients in the pre‐ and postchemotherapy setting.^4^, ^5^ Unfortunately, many patients show intrinsic or develop acquired resistance to ENZA therapy.

In recent years, different ENZA resistance mechanisms have been discovered,^11^ however the molecular background of these has still not been completely elucidated. One of the potential key mechanisms of ENZA resistance is associated with AR alterations, including AR amplification, AR point mutations and expression of constitutively active AR splice variants.^11^, ^12^, ^13^ In addition, non‐AR related resistance mechanisms have been shown to be involved in ENZA resistance. Elevated levels of neuroendocrine serum markers, such as chromogranin A and neuron‐specific enolase were independently associated with ENZA‐treated patients' shorter survival,^14^ while this correlation was less obvious in DOC‐treated patients.^15^ Additionally, several studies revealed correlations between DNA repair‐ (BRCA, ATM, MMR), Wnt‐pathway genes alterations and ENZA resistance.^11^, ^16^, ^17^, ^18^ Moreover, TP53 gene defect and RB1 loss were associated with resistance against next generation AR inhibitors.^16^, ^19^ We recently found that elevated baseline MMP‐7 levels are independently associated with resistance to ENZA therapy in CRPC patients.^20^ Despite promising results, none of these biomarkers have been incorporated in clinical decision‐making.

In this study, our aim was to uncover the mechanisms and potential biomarkers for ENZA resistance. For this, we used a comparative proteome analysis to identify differentially expressed proteins between ENZA‐resistant vs ENZA‐sensitive parental cell lines. Then, significantly upregulated proteins in ENZA‐resistant cells were filtered by different methods to select the most promising candidates. Selected proteins were assessed in serum samples of ENZA‐treated mCRPC patients by using the ELISA method. Serum concentrations were correlated with clinicopathological and follow‐up data. As ALCAM (activated leukocyte cell adhesion molecule) was the only serum protein to be associated with survival in ENZA‐treated patients, its serum levels were determined also in ABI‐ and DOC‐treated mCRPC patients. Finally, in order to evaluate its functional involvement in ENZA resistance, we performed siRNA‐mediated ALCAM knockdown analyses in cell lines and determined its effect on ENZA sensitivity.

## 2 MATERIALS AND METHODS

### 2.1 Cell culture and reagents

Enzalutamide sensitive LAPC4 (RRID:CVCL_4744), DuCaP (RRID:CVCL_2025) and resistant LAPC4‐ENZA and DuCaP‐ENZA PC cell lines were a gift from Dr. Martin Puhr. ENZA resistance has been developed by the dose escalation method as described by Hoefer et al.^21^ LAPC4 and DuCaP cells were grown in RPMI 1640 medium supplemented with 10% fetal bovine serum (Biowest, Nuaillé, France), 1% Glutamax (Thermo Fisher Scientific) and 1% penicillin/streptomycin (Lonza, Basel, Switzerland). LAPC4 were further supplemented with 1 nM dihydrotestosterone (DHT). All cells were maintained at 37°C in 5% CO2 with humidified atmosphere. Medium was changed every third day. The parental cell lines as well as the ENZA‐resistant subclones have recently been authenticated using short tandem repeat profiling (June 2021). All experiments were performed with mycoplasma‐free cells. ENZA (MedChemExpress) was dissolved in DMSO as a 100 mM stock solution and stored at −80°C.

### 2.2 Liquid chromatography tandem mass spectrometry (LC‐MS/MS) analysis

Proteome analyses were performed using the LC‐MS/MS technique in order to identify differentially expressed proteins between ENZA‐sensitive (LAPC4, DuCaP) and ENZA‐resistant (LAPC4‐ENZA, DuCaP‐ENZA) cell lines. For the LC‐MS/MS analysis, six technical replicates for each cell lines were used. The significance of expression changes was tested by calculating an unpaired, Students' *t* test. Details on sample preparation, LC‐MS/MS and protein identification are described in Supplementary Materials and Methods.

### 2.3 Gene ontology and KEGG pathway analysis

Gene Ontology (GO) biological process annotation and KEGG enrichment analysis was performed with using STRING (string-db.org). Proteins that were significantly up‐ and downregulated in ENZA‐resistant cells, respectively, were analyzed separately. The enriched ontologies (Biological Process) were filtered (observed gene count ≥25, false discovery rate ≤0.0001, strength ≥0.25, background gene count ≤3000) and manually reviewed to remove redundant and inapplicably terms.

### 2.4 Biomarker selection

In ENZA‐resistant cells, proteins quantified by at least two unique peptides with significantly higher abundance than those of ENZA‐sensitive cells at *t*‐test *P* < .05 were considered as significantly differentially abundant (Figure S1). In order to identify the most promising proteins, we used three different approaches.

First, in order to identify potentially secreted proteins, we used a workflow, which applies the combination of existing prediction programs (SignalP 4.1, SecretomeP 2.0, TargetP 1.1 and TMHMM 2.0) and databases (Uniprot, Human Protein Atlas, NCBI, ExoCarta).

Second, we performed a cross‐reference analysis with a formerly published independent transcriptome dataset of ENZA‐sensitive and ‐resistant prostate cancer cell lines in order to identify an overlap with our list of upregulated proteins.^22^

Third, we applied a pathway‐ and literature‐based selection method by scoring molecular interactions (number of edges) according to the STRING database. In addition, we considered the proteins' known oncological role according to literature as well as the availability of ELISA assays for later serum analyses.

### 2.5 Patient cohort and sample

Pretreatment serum concentrations of selected proteins were measured in samples of 72 mCRPC patients who were treated with ENZA therapy as first or later line treatment between September 2013 and March 2016. As we found high baseline ALCAM levels to be associated with poor survival of ENZA‐treated patients, we measured ALCAM concentrations also in baseline serum samples of ABI‐ and DOC‐treated mCRPC patients. The ABI cohort included 101 mCRPC patients who underwent ABI therapy between November 2008 and May 2015. The DOC cohort included 100 mCRPC patients who received DOC chemotherapy between January 2013 and April 2019. Serum samples were collected directly before commencing ENZA, ABI and DOC treatment. In addition, for 64 ENZA‐, 39 ABI‐ and 44 DOC‐treated patients, serum samples at 3 months after initiation of therapy start were also available. Serum samples were collected at the Department of Urology at the Medical University of Vienna and at the Semmelweis University, Budapest. Blood samples were collected in 9 mL Vacuette tubes (Greiner Bio‐One GmbH, Frickenhausen, Germany) and centrifuged for 10 min at room temperature with an Eppendorf 5702R centrifuge at 1500*g* within 3 h of venepuncture. Serum samples were immediately aliquoted and stored on −80°C until analysis. The study was performed in accordance with the ethical standards of the Helsinki Declaration and was approved by the ethical boards of the hospitals (TUKEB 55/2014, ECS 1986/2017). PSA response was defined, according to the Prostate Cancer Clinical Trials Working Group Criteria (PCWG) II, as at least 50% PSA decline from baseline during therapy.^23^

### 2.6 Serum ELISA analyses

ALCAM serum levels were quantified in 420 serum samples of 273 patients by using the DuoSet ELISA assay (R&D Systems, Minneapolis, MN) according to the manufacturer's instructions. Serum concentration of AGR2, IDH1 and NDRG1 were measured in 72 ENZA‐treated patients by using ELISA assays (AGR2: SEC285Hu, IDH1: SEH839Hu, Cloud Clone Corp, USA; NDRG1: OKEH02359, Aviva System Biology Corp, San Diego; respectively). Absorbance was measured at 450 nm by a Multiscan FC Microplate Photometer (Thermo Fisher Scientific, Darmstadt, Germany).

### 2.7 Western blot analysis

For Western Blot analysis, cells were lysed in RIPA Lysis and Extraction Buffer (Thermo Fisher Scientific) containing 1:100 Halt Protease Inhibitor Cocktail (Thermo Fisher Scientific) and Halt Phosphatese Inhibitor Cocktail (Thermo Fisher Scientific) according to the manufacturer's instructions. A total of 20 μg protein per sample was loaded on bolt 4% to 12% Bis‐Tris Plus gels (Thermo Fisher Scientific) for electrophoresis separation. Then, gels were blotted onto nitrocellulose membrane (iBlot 2 Transfer Stacks, Thermo Fisher Scientific) using iBlot 2 Gel Transfer Device. Gel electrophoresis and transfer was performed according to the manufacturer's instructions. Membranes were incubated in 5% nonfat dry milk (NFDM)/Tris‐buffered saline with 0.1% Tween 20 detergent (TBST) for 1 h at room temperature. The following antibodies were used: anti‐ALCAM antibody (1:10.000; Abcam, ab109215) with goat anti‐rabbit secondary antibody (1:4.000; Abcam, ab6721); anti‐GAPDH antibody (1:8000; Abcam, ab8245) with goat anti‐mouse secondary antibody (1:6000, Abcam, ab6789). Antibody binding on the membrane were detected by SuperSignal West Pico Chemiluminescent Substrate Kit (Thermo Fisher Scientific) and visualized on iBright FL1000 Imaging System (Thermo Fisher Scientific). To quantify protein relative amounts we used ImageJ software.^24^

### 2.8 Viability assay

Cell viability was detected by WST‐1 assay (Roche) according to the manufacturer's protocol. 3500 cells per well were seeded in 100 μL medium onto 96 well plate. On the next day, the medium was changed to fresh medium containing the indicated concentrations of ENZA. On day 3, the old medium was replaced by 100 μL of fresh medium containing the appropriate amounts of ENZA. Cells were maintained at 37°C in 5% CO^2^ with humidified atmosphere. On the day 6, 10 μL of WST‐1 solution was added into each well and cells were incubated for 4 h at 37°C with 5% CO^2^. Measurements were done in at least three independent biological experiments with three technical replicates.

### 2.9 siRNA silencing

As ALCAM was not detectable either in DuCaP or in DuCaP‐ENZA cell lines, siRNA‐mediated ALCAM silencing was only performed in LAPC4 and LAPC4‐ENZA cells. Cells were seeded in six well plate in 2 mL antibiotic‐free RPMI‐1640 medium, then transfected with 40 pmol siRNA using Lipofectamine RNAiMAX Transfection Reagent (Invitrogen, 13 778 150) and Opti‐MEM Reduced Serum Medium (Gibco). We used SMARTpool on‐target plus siRNA against ALCAM (Dharmacon, Lafayette, CO, Cat. No: L‐004574‐00‐0005) and nontargeting control pool on‐target plus siRNA (Dharmacon, D‐0018101005). Medium was changed 8 h after transfection. Gene silencing was monitored by Western blot.

### 2.10 Real‐time quantitative PCR

Details of RT‐qPCR are described in Supplementary Materials and Methods.

### 2.11 In silico analysis of ALCAM gene expression

In silico analysis was performed by using TNMplot (http://www.tnmplot.com) and cBioPortal databases (https://www.cbioportal.org). TNMplot database were used to evaluate the expression of ALCAM in various cancers and to explore the ALCAM mRNA expression in normal, tumor and metastatic PC tissues.^25^ In order to analyze the correlation between ALCAM expression and clinicopathological data, we extracted mRNA expression data of 266 mCRPC patients and their genomic and clinical information from the TCGA using cBioPortal database.^26^, ^27^

### 2.12 Statistical analysis

In the case of ELISAs, statistical analyses were done with the SPSS 26.0 (IBM, Chicago, Illinois) software. For independent group comparisons, the nonparametric two‐sided Wilcoxon rank sum test (Mann‐Whitney test) was used. Spearman's and Pearson's correlation were used for measure the degree of correlations between ALCAM serum levels and continuous clinical and laboratory parameters. Univariable survival analyses were done using both Kaplan‐Meier curves with log‐rank tests and univariable Cox regression analysis. For multivariable analysis, Cox proportional hazards regression modeling was used with parameters that were statistically significant in univariable analysis at *P* < .05. To determine the optimal cut‐off value with the highest sensitivity and specificity for the prediction of death within 24 months, we used the nonparametric receiver operating characteristics (ROC) curves in which the value for sensitivity is plotted against false‐positive rate (1‐specificity). The concentrations with the highest specificity and sensitivity values (Youden index) were defined as optimal cut‐offs. IC50 values were calculated by using GraphPad Prism 8 (GraphPad Software Inc., La Jolla, California). In all tests, *P* values <.05 were considered statistically significant.

## 3 RESULTS

### 3.1 Identification of proteins with differential expression between ENZA‐sensitive and ‐resistant PC cells

**FIGURE 1:**
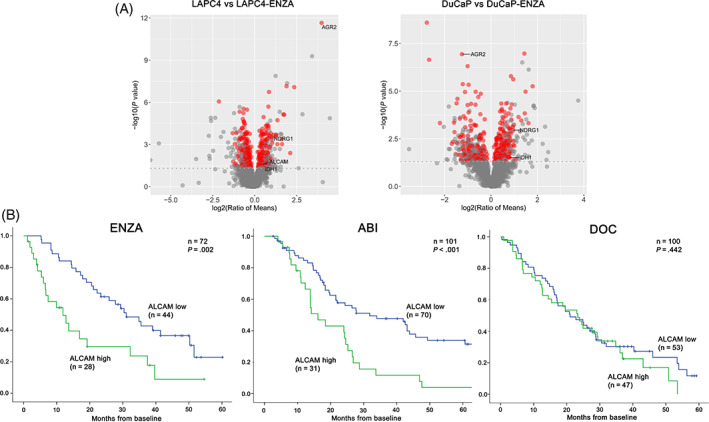
(A) Volcano plot visualization of detected proteins (as dots) by LC‐MS/MS analysis of LAPC4 vs LAPC4‐ENZA and DuCaP vs DuCaP‐ENZA cell lines. The red dots represent the significantly higher abundant proteins in cells. (B) Kaplan‐Meier overall survival curves show that high ALCAM baseline serum levels are associated with poor OS in ENZA‐ (left), ABI‐ (middle) but not in DOC‐ (right) treated patients [Color figure can be viewed at wileyonlinelibrary.com]

LC‐MS/MS proteome analyses revealed 385 (LAPC4 vs LAPC4‐ENZA) and 346 (DuCaP vs DuCaP‐ENZA) significantly differentially abundant (either increased or decreased) proteins in cells (Figures 1,A and S1 and Tables S1 and S2). We focused on those proteins that were detected by at least two unique peptides and were significantly upregulated in ENZA‐resistant cells (Figure S1).

Using the first selection method, we identified five (AGR2, PFKP, APOD, ALCAM and DYNLT3) potentially secreted proteins.

Based on cross‐reference analysis, we identified seven proteins (GALK2, PTGR1, SCIN, IDH1, AHCY, THOP1 and SF3B5).

Our third, pathway‐ and literature‐based selection method identified four proteins (AGR2, RHOA, NDRG1 and ALCAM).

Finally, identified targets were filtered by the availability of ELISA assays which selected four proteins (ALCAM, AGR2, IDH1 and NDRG1) for serum analyses.

### 3.2 GO and KEGG pathway analysis

GO analysis found the highest number of enriched categories for proteins upregulated in LAPC4 cells (Figure S2). However, the most significantly enriched ontologies were corresponding to those proteins downregulated in LAPC4. For DuCaP more enriched categories were correspond to upregulated proteins although the numbers of analyzed proteins were not much different between up‐ and downregulated fractions. Some categories were homogeneously enriched for several of the analyzed fractions, such as ontologies associated with the metabolism of nucleic acids or its chemical synonyms. Accordingly, processes associated with gene expression and RNA processing were also significantly enriched.

Enrichment analysis of KEGG pathways resulted in complementary findings to GO analysis (Figure S3). Most enriched terms were found for proteins upregulated in LAPC4 (compared with downregulated ones for the gene ontology analysis). However, the most significant KEGG pathway was again found for proteins downregulated in LAPC4 (ie, Ribosome) and corresponded to processes associated to protein translation. Proteins upregulated in LAPC4 were associated with diverse metabolic pathways, such as carbon metabolism and amino acid metabolism. For DuCaP cells, metabolic pathways were enriched for both, up and downregulated proteins. Furthermore, upregulated proteins were enriched in pathways related to DNA replication and RNA processing, which was congruent with enriched gene ontologies.

### 3.3 Selected protein levels in patients' samples

#### 3.3.1 Patients' characteristics

The median age in the ENZA cohort was 73 years (range: 56‐89), the median pretreatment PSA value was 69.5 ng/mL. The primary end‐points of the analysis was overall survival (OS). 48 (66%) patients died during a median follow‐up period of 19 months. 1‐year survival was 65%, 2‐years survival was 43% (Table 1).

**TABLE 1:** Patients' characteristics in patients who underwent ENZA, ABI and DOC treatment

Parameters	ENZA	ABI	DOC
Baseline characteristics			
Total No. of patients	72	101	100
Age (median)	73 (56–89)	71 (54‐90)	70 (43‐86)
PSA (median; ng/mL)	69.48 (0.23‐8422.0)	65.30 (0.10‐6785.0)	88.77 (3.20‐6115.41)
LDH (median; U/I)	200.0 (117.0‐4930.0)	212.5 (100.0‐1180.0)	261.0 (143.0‐1123.0)
AP (median; U/I)	92.0 (32.0‐591.0)	93.0 (35.0‐4149.0)	224.0 (55.0‐1204.0)
CRP (median; mg/L)	0.6 (0.02‐10.8)	0.4 (0.02‐14.2)	6.5 (0.4‐264.1)
Hemoglobin (median; g/dL)	12.1 (6.2‐168.0)	11.7 (7.9‐106.0)	12.95 (8.3‐15.2)
ECOG PS (%) 0	49 (68)	57 (56)	65 (65)
1	10 (14)	16 (16)	26 (26)
2	4 (6)	0	9 (9)
Unknown	9 (12)	28 (28)	0
Pain (%)			
Yes	19 (26)	36 (35)	—
No	46 (64)	50 (50)	—
Unknown	7 (10)	15 (15)	100 (100)
Metastases (%)			
Bone	61 (85)	89 (89)	94 (94)
LN (>2 cm)	16 (22)	12 (12)	38 (38)
Visceral	5 (7)	9 (9)	13 (13)
Primary local therapy (%)			
Prostatectomy	29 (40)	48 (48)	18 (18)
Radiation	17 (24)	21 (21)	12 (12)
Therapy line (%)			
First line	15 (20)	38 (37)	97 (97)
Second or later line	57 (80)	60 (60)	3 (3)
Unknown	0	3 (3)	0
Follow‐up characteristics			
Max. PSA decline^a^ (%) >30	50 (69)	73 (73)	55 (55)
>50	47 (65)	65 (65)	45 (45)
>90	26 (36)	33 (33)	26 (26)
Subsequent therapy (%)			
No	33 (46)	49 (49)	51 (51)
DOC	5 (7)	15 (15)	—
ABI	5 (7)	—	19 (19)
ENZA	—	33 (33)	26 (26)
CABA	25 (35)	28 (28)	7 (7)
Xofigo	11 (15)	10 (10)	6 (6)
Lu‐PSMA	6 (8)	8 (8)	0
Number of patients died (%)	48 (66)	69 (69)	78 (78)
OS median, months	19.9	21.6	22.9

Abbreviations: ABI, abiraterone; AP, alkaline phosphatase; CABA, cabazitaxel; CRP, C reactive protein; ENZA, enzalutamide; DOC, docetaxel; LDH, lactate dehydrogenase; LN, lymph node; Lu‐PSMA, lutetium‐prostate‐specific membrane antigen; OS, overall survival; PS, performance status.

^a^ In comparison to baseline PSA during the given therapy.

In the ABI cohort, the median patients age was 71 years (range: 54‐90), the median PSA value was 65.3 ng/mL. In this cohort 69 (68%) patients died during a median follow‐up period of 21 months (Table 1).

The median age in DOC cohort was 70 years (range: 43‐86), the median PSA value was 88.8 ng/mL. Seventy‐eight (78%) of 100 DOC‐treated patients died during a median follow‐up time of 23 months (Table 1).

#### 3.3.2 Correlations of clinicopathological parameters with serum levels

We found no significant association between the assessed marker levels and patients' age, primary local therapy or PSA response (Tables 2 and S3 and S4). In the ENZA cohort, IDH1 levels were decreased in bone metastatic cases (*P* = .033), while AGR2 levels were lower in visceral metastatic patients (*P* = .010). NDRG1 serum levels were significantly lower in men who had no PSA response (*P* = .023). ALCAM serum levels were significantly higher in patients who had high baseline PSA, LDH, AP and CRP levels (*P* < .05), while it were significantly lower in patients responded with at least 30% PSA response to ENZA (*P* = .045; Table 2). In the ABI cohort, ALCAM levels were higher in patients who had pain (*P* = .013) and had high PSA, LDH and AP levels at baseline (*P* < .05; Tables 2 and S4). In the DOC and ABI cohort, ALCAM serum levels were significantly higher in men with poor ECOG status (*P* = .015 and *P* = .007; respectively). In all three therapy groups, Pearson's and Spearman's correlation analyses revealed a significant correlation between baseline ALCAM and PSA levels (Pearson *P* < .001; Spearman *P* < .001; Table S5).

**TABLE 2:** Association of baseline ALCAM levels with clinicopathological parameters in ENZA, ABI and DOC‐treated patients

	ENZA			ABI			DOC		
	n	ALCAM serum cc. (ng/mL)		n	ALCAM serum cc. (ng/mL)		n	ALCAM serum cc. (ng/mL)	
	72	Median (range)	*P*	101	Median (range)	*P*	100	Median (range)	*P*
Whole cohort	72	127.8 (0‐400.0)		101	120.5 (45.2‐400.0)		100	114.2 (47.2‐400.0)	
Age (years)									
≤72	35	136.1 (82.3‐400.0)	.673	56	121.4 (45.2‐389.2)	.830	54	120.7 (51.9‐400.0)	.582
>72	37	123.6 (0‐400.0)		45	120.5 (55.1‐400.0)		46	113.0 (47.2‐219.8)	
Primary therapy									
No	28	133.7 (82.5‐400.0)	.677	34	131.9 (55.1‐400.0)	.556	71	129.0 (51.9‐400.0)	.099
Yes	44	127.4 (0‐400.0)		67	119.9 (45.2‐389.2)		29	107.4 (47.2‐219.8)	
Line of above therapy									
1st line	15	117.0 (82.3‐400.0)	.261	38	131.9 (45.2‐400.0)	.572	97	113.9 (47.2‐400.0)	.564
2nd or later line	57	136.1 (0‐400.0)		60	118.8 (49.6‐389.2)		3	135.0 (60.5‐400.0)	
Unknown	0			3			0		
ECOG PS									
0	49	132.3 (0‐400.0)	.785	57	115.1 (45.2‐389.2)	**.007**	65	107.0 (47.2‐315.9)	**.015**
1‐2	14	122.4 (83.04‐367.9)		16	144.2 (56.8‐400.0)		35	135.0 (60.4‐400.0)	
Unknown	9			28			0		
LN status									
N−	56	131.9 (0‐400.0)	.813	85	121.2 (45.2‐400.0)	.919	62	113.0 (47.2‐400.0)	.867
N+	16	124.350 ( 92.2‐256.1)		15	118.1 (95.4‐198.8)		38	126.7 (51.8‐400.0)	
Unknown	0			1					
Visceral mets.									
No	67	128.0 (0‐400.0)	.450	90	120.3 (45.2‐400.0)	.817	87	111.2 (47.2‐219.8)	.056
Yes	5	114.2 (83.5‐153.9)		9	136.4 (75.1‐386.8)		13	153.7 (67.9‐242.8)	
Unknown	0			2					
Bone mets.									
No	11	114.6 (88.0‐171.4)	.133	11	114.4 (56.8‐138.9)	.062	6	100.9 (64.9‐219.8)	.384
Yes	61	136.1 (0‐400.0)		89	122.6 (45.2‐400.0)		94	116.1 (47.2‐400.0)	
Unknown	0			1			0		
PSA baseline									
High*	36	154.2 (82.5‐400.0)	**<.001**	50	139.5 (52.9‐400.0)	**.016**	50	156.7 (57.6‐400.0)	**.016**
Low*	36	110.7 (0‐208.6)		51	114.9 (45.2‐286.0)		50	105.9 (47.2‐240.3)	
Unknown	0			0			0		
LDH									
>240 U/L	23	157.3 (84.3‐400.0)	**.001**	35	141.1 (67.9‐400.0)	**.010**	15	151.2 (51.9‐282.2)	.167
<240 U/L	46	117.5 (0‐400.0)		65	116.9 (45.2‐291.1)		12	105.5 (63.9‐239.5)	
Unknown	3			1			73		
AP									
>129 U/L	22	157.1 (83.9‐400.0)	**.008**	41	144.4 (52.9‐389.2)	**.001**	21	133.9 (51.9‐282.2)	.492
<129 U/L	47	119.5 (0‐400.0)		58	115.0 (45.2‐400.0)		10	111.8 (67.6‐239.5)	
Unknown	3			2			69		
CRP									
>5 mg/L	7	184.3 (127.5‐400.0)	**.012**	12	145.6 (68.8‐198.8)	.367	18	136.5 (51.9‐282.2)	.075
<5 mg/L	61	121.0 (0‐400.0)		88	120.0 (45.2‐400.0)		13	97.0 (57.6‐223.2)	
Unknown	4			1			69		
Hemoglobin									
>13.5 g/dL	9	114.6 (100.2‐400.0)	.334	23	111.7 (55.1‐291.1)	.253	8	105.5 (51.9‐282.2)	.170
<13.5 g/dL	61	135.9 (0‐400.0)		76	122.5 (45.2‐400.0)		22	146.0 (67.6‐2382.2)	
Unknown	2			2			70		
PSA decline									
>30%	50	121.9 (0‐400.0)	**.045**	73	119.9 (45.2‐400.0)	.322	55	104.8 (57.6‐400.0)	.910
<30%	22	147.6 (68.3‐400.0)		28	121.8 (55.1‐400.0)		31	129.0 (47.2‐400.0)	
Unknown	0			0			14		

*Note*: Significant values are indicated in bold. P‐value was calculated with the Mann‐Whitney *U* test. For LDH, AP, CRP and Hemoglobin the upper value of routine clinically defined normal ranges were used as cut‐off. *ENZA cohort: PSA: 69.45 ng/mL (median); *ABI cohort: PSA: 65.3 ng/mL (median); *DOC cohort: PSA: 93.85 ng/mL (median).

Abbreviations: AP, alkaline phosphatase; CRP, C reactive protein; LDH, lactate dehydrogenase; LN, lymph node; PS, performance status.

#### 3.3.3 ROC analysis

For ROC analysis, OS were dichotomized at 24th month of survival. ROC analysis identified 146.6 ng/mL as an optimal cut‐off value with the highest sensitivity (58.8%) and specificity (68.4%) for ALCAM in ENZA cohort. In ABI cohort ROC method found the cut‐off value with the highest sensitivity (37.2%) and specificity (89.3%), which resulted 143.1 ng/mL as an optimal cut‐off. In the ENZA&ABI cohort ROC analysis identified 137.7 ng/mL as an optimal cut‐off value with the highest sensitivity (51.9%) and specificity (70.7%). In the DOC cohort, ROC method found 127.9 ng/mL as an optimal cut‐off value with the highest sensitivity (46.2%) and specificity (59.1%). These cut‐off values were further analyzed in subsequent survival analyses.

#### 3.3.4 Survival analyses

In the ENZA cohort, presence of bone or visceral metastases were associated with shorter OS (*P* = .004 and *P* = .047; respectively; Table 3). Patients who underwent second‐ or later‐line of ENZA therapy had significantly worse survival (*P* = .021; Table 3). High PSA and ALCAM pretreatment serum levels were significantly associated with shorter OS (*P* < .05; Table 3).

**TABLE 3:** Univariable analysis in patients who underwent ENZA, ABI, ENZA&ABI and DOC treatment

Variables		ENZA			ABI			ENZA&ABI			DOC		
		Overall survival			Overall survival			Overall survival			Overall survival		
		HR	95% CI	*P*	HR	95% CI	*P*	HR	95% CI	*P*	HR	95% CI	*P*
Age	>72 years	1.038	0.589‐1.837	.898	1.612	0.984‐2.641	.058	1.335	0.921‐1.934	.127	1.204	0.768‐1.886	.419
ECOG	2	1.357	0.638‐2.886	.428	3.970	2.070‐7.614	**<.001**	1.601	0.504‐5.087	.425	2.128	1.349‐3.357	**.001**
Visceral mets.	pos.	3.511	1.017‐12.122	**.047**	1.731	0.788‐3.803	.171	0.912	0.365‐2.280	.844	1.567	0.860‐2.856	.143
LN mets.	pos.	0.622	0.290‐1.330	.221	1.627	0.864‐3.131	.145	1.026	0.626‐1.680	.920	0.942	0.592‐1.498	.800
Bone mets.	pos.	5.796	1.773‐18.945	**.004**	2.050	0.821‐5.116	.124	1.746	1.199‐2.541	**.004**	1.260	0.396‐4.011	.696
Primary RPE	pos.	0.779	0.512‐1.651	.919	0.775	0.481‐1.250	.296	0.822	0.569‐1.188	.296	1.144	0.648‐2.017	.643
Primary RAD	pos.	0.661	0.329‐1.328	.245	0.667	0.369‐1.242	.208	0.668	0.423‐1.056	.084	1.280	0.636‐2.577	.489
Line of therapy	2nd or ltr.	2.769	1.169‐6.559	**.021**	0.962	0.590‐1.571	.878	1.209	0.970‐1.507	.091	0.975	0.238‐3.990	.972
PSA median	*	3.696	2.036‐6.709	**<.001**	2.520	1.532‐4.144	**<.001**	2.986	2.039‐4.373	**<.001**	1.618	1.022‐2.563	**.040**
PSA decline	Yes	0.608	0.291‐1.269	.185	1.071	0.512‐2.240	.856	0.886	0.529‐1.484	.646	0.262	0.142‐0.485	**<.001**
PSA decline	>30%	0.740	0.385‐1.419	.364	0.560	0.317‐0.990	**.046**	0.623	0.407‐0.953	**.029**	0.467	0.286‐0.764	**.002**
PSA decline	>50%	0.735	0.394‐1.373	.335	0.555	0.327‐0.943	**.029**	0.626	0.419‐0.934	**.022**	0.521	0.319‐0.852	**.009**
PSA decline	>90%	0.471	0.255‐0.870	**.016**	0.623	0.375‐1.036	.068	0.559	0.379‐0.826	**.003**	0.561	0.318‐0.989	**.046**
ALCAM median	*	2.002	1.124‐3.564	**.018**	2.093	1.291‐3.395	**.003**	2.047	1.415‐2.962	**<.001**	1.483	0.940‐2.341	.091
ALCAM (ROC)	*	2.449	1.390‐4.491	**.002**	2.516	1.523‐4.157	**<.001**	2.658	1.883‐3.856	**<.001**	1.415	0.897‐2.231	.136
LDH	>240 U/L	2.945	1.599‐5.424	**.001**	2.173	1.315‐3.593	**.002**	2.477	1.683‐3.645	**<.001**	3.425	1.186‐9.891	**.023**
AP	>129 U/L	3.822	2.051‐7.121	**<.001**	2.892	1.736‐4.818	**<.001**	3.171	2.146‐4.687	**<.001**	1.802	0.710‐4.575	.215
CRP	>5 g/mL	3.268	1.222‐8.737	**.018**	2.448	1.196‐5.013	**.014**	2.700	1.521‐4.792	**<.001**	2.072	0.919‐4.673	.079
Hemoglobin	>13.5 g/dL	0.398	0.142‐1.117	.080	0.522	0.288‐0.944	**.031**	0.479	0.288‐0.796	**.005**	0.312	0.105‐0.928	**.036**
AGR2 median	>439.15 pg/mL	0.818	0.463‐1.444	.488	—	—	—	—	—	—	—	—	—
AGR2 (ROC)	>271.6 pg/mL	0.658	0.360‐1.201	.172	—	—	—	—	—	—	—	—	—
IDH1 median	>2694.0 ng/mL	0.816	0.416‐1.442	.484	—	—	—	—	—	—	—	—	—
IDH1 (ROC)	>2877.0 ng/mL	1.243	0.700‐2.205	.458	—	—	—	—	—	—	—	—	—
NDRG1 median	>25800.0 ng/mL	1.083	0.609‐1.926	.786	—	—	—	—	—	—	—	—	—
NDRG1 (ROC)	>31920.0 ng/mL	0.710	0.383‐1.316	.277	—	—	—	—	—	—	—	—	—

*Note*: Significant values are indicated in bold. *ENZA cohort: median (ng/mL) PSA: 69.45, ALCAM: 127.8/ALCAM ROC cut‐off (ng/mL): 146.6; *ABI cohort: median (ng/mL) PSA: 65.3, ALCAM: 120.5/ALCAM ROC cut‐off (ng/mL): 143.1; *ABI&ENZA cohort: median (ng/mL) PSA: 66.6, ALCAM: 122.4/ALCAM ROC cut off (ng/mL): 137.8; *DOC cohort: median (ng/mL) PSA: 88.02, ALCAM: 114.2/ALCAM ROC cut off (ng/mL): 127.95.

Abbreviations: AP, Alkaline phosphatase; CRP, C reactive protein; LN, lymph node; LDH, lactate dehydrogenase; RAD, radiation; RPE, radical prostatectomy.

In the ABI cohort, higher ECOG status, high baseline PSA and ALCAM serum levels were significantly associated with worse survival (*P* < .005; Table 3).

When combining the ENZA/ABI cohort, we found the presence of bone metastases, high PSA, LDH, AP, CRP, Hemoglobin and ALCAM pre‐treatment serum levels to be significantly associated with shorter OS (*P* ≤ .005; Table 3). In multivariable analysis, presence of bone metastases, high PSA, AP and ALCAM (>137.8 ng/mL) levels were independently associated with OS (*P* < .005; Table 4). In the ENZA&ABI first line treated subgroup, high baseline ALCAM levels were significantly associated with poor OS (Figure S4). Moreover, multivariable analysis identified high PSA and ALCAM (<131.9 ng/mL) levels as independent predictors of OS (*P* = .041 and *P* = .002; respectively; Table 4).

**TABLE 4:** Multivariable analysis in patients who underwent ENZA&ABI treatment in any treatment lines and in first line treated patients

ENZA&ABI cohort	Overall survival		
	HR	95% CI	*P*
**Any treatment lines**			
Bone mets.	1.859	1.242‐2.781	**.003**
PSA (median) > 66.6 ng/mL	2.404	1.580‐3.658	**<.001**
ALCAM (median) > 122.4 ng/mL	1.423	0.942‐2.150	.094
LDH > 240 U/L	1.269	0.795‐2.023	.318
CRP > 5 g/mL	1.616	0.852‐3.065	.141
AP > 129 U/L	2.065	1.311‐3.251	**.002**
Hemoglobin >13.5 g/dL	0.743	0.418‐1.320	.311
Bone mets.	1.834	1.224‐2.748	**.003**
PSA (median) > 66.6 ng/mL	2.372	1.560‐3.606	<**.001**
ALCAM (ROC) > 137.8 ng/mL	1.833	1.213‐2.771	**.004**
LDH > 240 U/L	1.205	0.754‐1.924	.436
CRP > 5 g/mL	1.600	0.851‐3.008	.144
AP > 129 U/L	2.014	1.274‐3.183	**.003**
Hemoglobin >13.5 g/dL	0.769	0.433‐1.368	.372
**First line treated patients**			
ALCAM (median) > 121.2 ng/mL	2.606	1.093‐6.213	**.031**
PSA (median) > 37 ng/mL	2.466	1.029‐5.907	**.043**
Hemoglobin >13.5 g/dL	0.486	0.188‐1.254	0.136
CRP > 5 g/mL	0.830	0.273‐2.529	.744
AP > 129 U/L	1.321	0.493‐3.539	.580
ALCAM (ROC) > 131.9 ng/mL	4.465	1.711‐11.651	**.002**
PSA (median) > 37 ng/mL	2.500	1.037‐6.030	**.041**
Hemoglobin >13.5 g/dL	0.508	0.202‐1.277	.150
CRP > 5 g/mL	0.735	0.244‐2.213	.584
AP > 129 U/L	0.911	0.327‐2.532	.857

*Note*: Significant values are indicated in bold.

Abbreviations: AP, alkaline phosphatase; CRP, C reactive protein; LDH, lactate dehydrogenase.

In the DOC cohort, ECOG status (>1) and high baseline PSA levels were associated with poor OS, while ALCAM showed no significant association with OS (Table 3).

In the ENZA and ABI cohort, Kaplan‐Meier OS curves revealed that higher baseline ALCAM levels (>146.6 and >143.1 ng/mL by ROC cut‐off) are significantly associated with shorter OS (*P* = .002 and *P* < .001; respectively; Figure 1B). Moreover, we combined pre‐treatment ALCAM and PSA serum levels and performed OS analysis by stratifying patients according to their received treatments (ENZA/ABI and DOC) in the whole cohort (n = 273). In addition, we performed subgroup analyses in first line treated patients and also in patients who received their last line of therapy. We found that in the low PSA and ALCAM levels group, ENZA/ABI‐treated men had a significant better OS compared with those who received DOC therapy regardless of treatment line (*P* ≤ .001; Figures S5, S6 and S7).

#### 3.3.5 Prognostic value of ALCAM levels changes during therapy

ALCAM level changes were calculated at 3 months after treatment start and were dichotomized as any increase, at least 30% and 50% increase. We found that patients with at least 30% increase in their ALCAM levels at 3 months had a significant shorter OS (ENZA cohort HR: 2.842; 95% CI 1.226‐6.585; *P* = .011, ABI cohort HR: 2.710; 95% CI 1.114‐6.595; *P* = .022; DOC cohort HR: 2.224; 95% CI 1.062‐4.661; *P* = .030; respectively).

### 3.4 Protein level of ALCAM in cell lines

**FIGURE 2:**
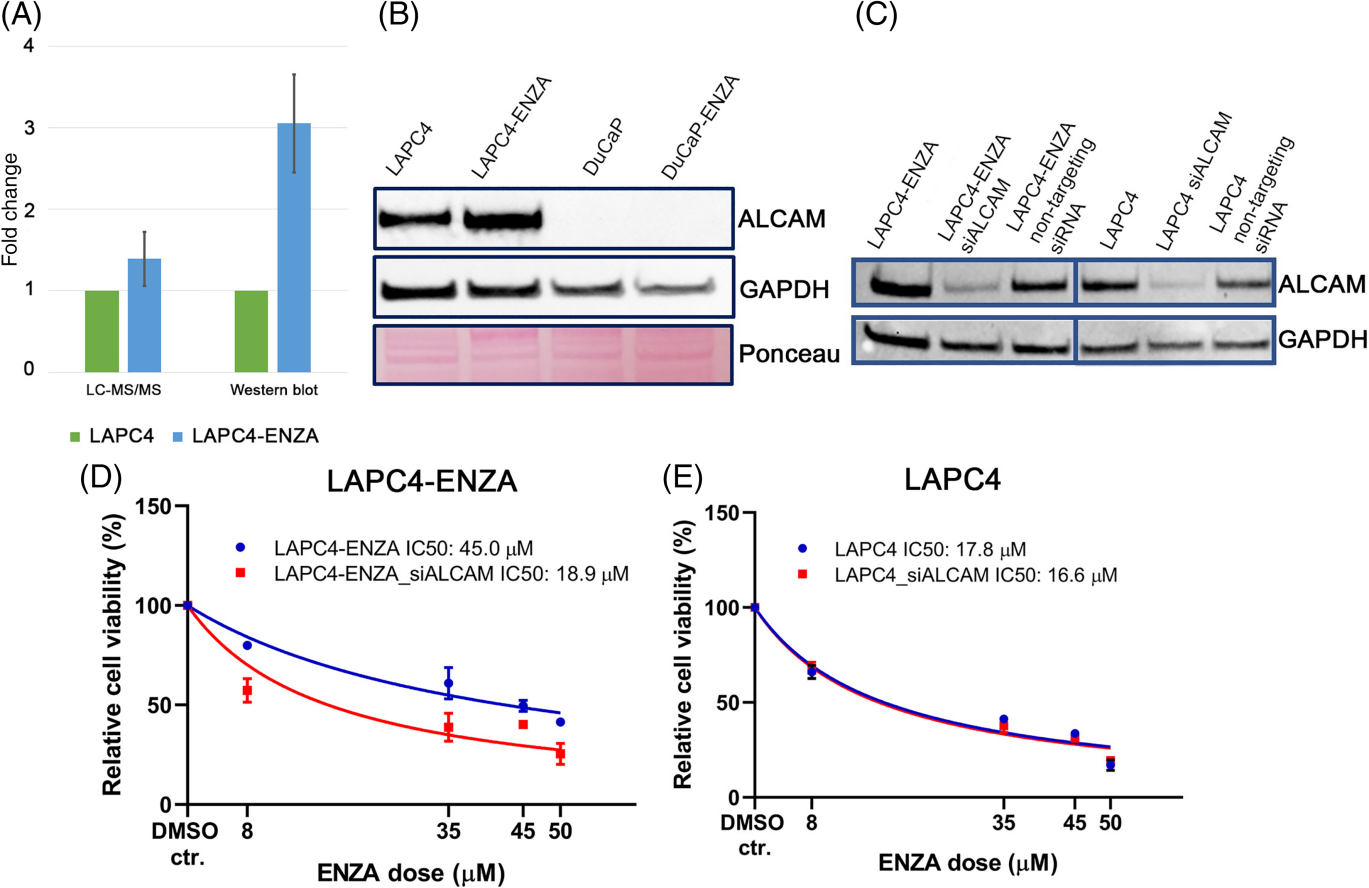
(A) Bar chart presentation of LC‐MS/MS and Western‐blot analyses of ALCAM expression in ENZA‐sensitive (LAPC4) and ‐resistant (LAPC4‐ENZA) cells. Data is presented as mean ± SD from three and six independent experiments for Western blot (by densitometry) and LC/MS‐MS analyses, respectively. (B) Western‐blot analysis demonstrated higher ALCAM expression in LAPC4‐ENZA cells compared with parental LAPC4 cells. In DuCaP cell lines no ALCAM expression were detected. GAPDH and Ponceau staining were used as internal references. (C) Western‐blot analysis for ALCAM protein expression after transient transfection with siALCAM siRNA or nontargeting siRNA (D) Cell viability after 6 days of exposure to different concentrations of ENZA in LAPC4‐ENZA and LAPC4 (E) cell lines were measured by WST assay. For each curve the cell viability was calculated relative to the DMSO solvent control treatment. IC50 of ENZA for LAPC4‐ENZA siALCAM cells was significantly lower (*P* = .007) (IC50 = 18.8 μM) than the IC50 for LAPC4‐ENZA (IC50 = 45.0 μM). Data were presented as mean ± SD from three independent experiments [Color figure can be viewed at wileyonlinelibrary.com]

Based on ELISA analyses, we selected ALCAM to assess its potential functional involvement in ENZA resistance. First, we compared ALCAM expressions between ENZA‐sensitive and ‐resistant cell lines at the protein level by Western‐blot. In line with our LC‐MS/MS data—which found 1.39‐fold higher expression of ALCAM in LAPC4‐ENZA cells (compared with LAPC4)—Western‐blot analysis found a 3.05‐fold upregulation of ALCAM (Figure 2A,B).

### 3.5 Functional analyses of ALCAM in PC cell lines

To investigate the functional role of ALCAM in ENZA resistance, we performed siRNA‐mediated knockdown analysis. The siRNA transfection successfully silenced expression of ALCAM in LAPC4‐ENZA and parental LAPC4 cells according to Western blot measurements (Figure 2C).

To examine ENZA treatment in ALCAM silenced cells, WST proliferation assay were performed with parental LAPC4 and LAPC4‐ENZA cell lines. We found that ALCAM knock‐down significantly enhanced ENZA‐sensitivity in LAPC4‐ENZA cells compared with the nontransfected cells (Figure 2D), while in the parental LAPC4 cells no similar effect could be observed (Figure 2E).

### 3.6 Effect of ALCAM knock‐down of expression of AR and their target genes in cell lines

To determine the possible effect of ALCAM knock‐down to mRNA expression of AR and their target genes, PSA, FKBP5 and c‐myc were measured using RT‐qPCR. We found that AR, PSA, c‐myc and FKBP5 were significantly upregulated in ENZA resistant LAPC4 cells compared with parental/ENZA sensitive cell lines. ALCAM knock‐down did not significantly influenced AR, PSA and c‐myc gene expression levels, while FKBP5 expression decreased significantly (Figure S8).

### 3.7 Gene expression level of ALCAM and correlation with clinical data in databases

Using the TNMplot database, we compared ALCAM gene expressions between normal and tumor tissues in several organs and found ALCAM expression to be significantly elevated in breast, lung, renal, thyroid and prostate cancers compared with corresponding nonmalignant tissues. Notably, we found the largest difference in ALCAM gene expression between normal and respective tumor tissues in prostate and breast cancers (Figure S9). In prostate cancer, the highest ALCAM expressions were found in metastatic cases (Figure S9). To investigate the correlation between ALCAM mRNA expression and clinicopathological and follow‐up data we applied the TCGA dataset by using cBioPortal. This analysis revealed no significant association between ALCAM expressions and survival of mCRPC patients. In addition, high ALCAM mRNA expression was significantly associated with higher AR gene expression but lower neuroendocrine (NEPC) gene expression scores and absence of small cell histological variants (Figure S9).

## 4 DISCUSSION

Several mechanisms and signaling pathways have been found to be implicated in ENZA resistance of PC, for example, the alterations of the AR, transdifferentiation to neuroendocrine tumors, glucocorticoid receptor overexpression, DNA‐repair and PI3K pathway alterations.^11^, ^12^, ^13^, ^14^, ^15^, ^16^, ^17^, ^19^ Despite our increasing understanding of ENZA resistance, currently no markers predictive of ENZA resistance are available in clinical practice. Moreover, likely more than one molecular mechanism is involved in ENZA resistance. Therefore, our aim was to uncover proteins potentially involved in ENZA resistance and to test their value in the prediction of response to ENZA treatment when measured in serum samples of mCRPC patients. Using proteome analysis and applying three different hypothesis‐free selection methods, we identified 14 candidate ENZA‐resistant proteins. Four of the most promising proteins were determined in baseline serum samples of ENZA‐treated mCRPC patients.

The first, “secreted protein” selection method identified ALCAM and Anterior gradient 2 (AGR2) proteins. AGR2 is a proto‐oncogene that plays a functional role in angiogenesis, cell migration, cell growth and differentiation.^28^ In different cancer types, high AGR2 expression was associated with poor patients' prognosis.^29^ The predictive value of AGR2 in PC was assessed for DOC and ENZA and revealed different roles for AGR2 between these therapies. Zhao et al^30^ showed that AGR2 is downregulated in DOC resistant cell lines and AGR2 silencing resulted in DOC resistance in PC3 cells, suggesting that AGR2 expressions are directly associated with DOC sensitivity. An opposite effect was observed for ENZA by Tang et al^31^ showing that inhibition of KDM1B caused AGR2 suppression which increased ENZA sensitivity of PC cells. Our present comparative proteome analysis showed conflicting results regarding the expression of AGR2; as it was strongly upregulated in LAPC4‐ENZA‐resistant cells, while it was significantly down regulated in DuCaP‐ENZA cell lines. In addition, our serum analyses did not reveal ARG2 as serum marker of ENZA resistance.

The second “cross‐reference” analysis compared our proteome results with a published transcriptomics dataset.^22^ Qian et al performed an integrative analysis of expression data of ENZA‐ sensitive and ‐resistant PC cell lines. Comparing their upregulated gene list with our proteome dataset, nine matching proteins could be identified. Of these, we selected isocitrate dehydrogenase 1 (IDH1) for ELISA analysis. IDH1 is a metabolic enzyme, which converts isocitrate to 2‐oxoglutarate. According to the TCGA (The Cancer Genome Atlas) study in PC, IDH1 mutation carrier tumors represent a distinct molecular subclass of primary PCs. These mutations are enriched in early onset PCs and are associated with DNA hypermethylation.^32^ The role of IDH1 in ENZA resistance has not been evaluated yet. Our serum IDH1 analyses in ENZA‐treated CRPC patients revealed lower IDH1 levels in bone metastatic patients but showed no associations with OS.

The third “pathway‐ and literature‐based” selection method identified NDRG1 and ALCAM proteins. N‐myc downstream regulated gene‐1 (NDRG1) plays a role in cell growth, differentiation, signal transduction and is known as a tumor suppressor in various types of cancer including PC. However, some studies suggested NDRG1 as a proto‐oncogene.^33^ In a gene expression analysis Zheng et al^34^ identified 12 hub genes including NDRG1 which are potentially associated with ENZA resistance, however its exact role in ENZA resistance has not been elucidated yet. In the present study, we found no correlation between NDRG1 serum levels and OS of ENZA‐treated patients.

Activated leukocyte cell adhesion molecule (ALCAM), also called CD166 is a 100 to 105 kDa transmembrane glycoprotein which is a member of the immunoglobin superfamily. ALCAM is an adhesion molecule, which can bind to the CD6 receptor resulting in a heterophilic interaction or can form an ALCAM‐ALCAM homophilic dimer. It is involved in cell‐cell adhesion, angiogenesis, cell migration, invasion as well as therapy resistance and tumor progression.^35^ Recent studies reported that high tissue expressions of ALCAM are correlated with poor survival in pancreatic, bladder and colorectal cancer.^36^, ^37^, ^38^ In contrast, other studies found decreased ALCAM expression to be associated with worse prognosis.^39^, ^40^ In PC, Kristiansen et al^41^ found that ALCAM is upregulated in low‐grade tumors compared with high‐grade PC. In accordance, Minner et al^42^ showed that ALCAM overexpression was associated with favorable tumor stage and reduced risk of biochemical recurrence. In contrast, a more recent study of PC tissue samples revealed that high cytoplasmic ALCAM expression is associated with shorter PSA‐relapse‐free survival after radical prostatectomy^43^ and Hansen et al^44^ showed that ALCAM gene expression was elevated in metastatic PC patients and was associated with poor survival. In addition, Sanders et al^45^ determined the serum ALCAM levels in samples of 239 PC patients and found higher ALCAM levels to be associated with higher Gleason‐score, T‐stage and PSA levels suggesting an association between increasing serum ALCAM concentrations during prostate cancer progression.

To address the molecular and clinical correlates of ALCAM in tissue samples, we conducted *in silico* analyses in published datasets comparing ALCAM mRNA levels between normal and tumor tissues in several organs and found the largest increase in prostate and breast cancers (Figure S9). In PC, the highest ALCAM expressions were found in metastatic cases (Figure S9). However, in mCRPC we found no significant association between ALCAM expressions and patients' survival. In addition, high ALCAM mRNA expression was significantly associated with higher AR but lower neuroendocrine (NEPC) gene expression scores and absence of small cell histological variants (Figure S9). This suggest that ALCAM is rather involved in the regulation of androgen signaling then in neuroendocrine differentiation. This notion may have clinical relevance suggesting that already established neuroendocrine serum markers such as chromogranin A and neuron‐specific enolase as covering different resistance mechanisms might be well combined with ALCAM for the prediction of ENZA and ABI treatment.^15^ On the other hand, the correlation of ALCAM expression with AR gene expression score seems to be in line with current observations made in triple negative breast cancer where high ALCAM tissue protein expressions were shown to be associated with higher AR protein expression.^48^ However, in our experiment ALCAM knock‐down did not consistently influenced the expression of AR regulated genes, suggesting that ALCAM is coregulated with these genes rather than being their regulator.

The role of ALCAM in resistance to systemic therapy is poorly understood. Immunohistochemical analysis showed that tamoxifen resistant breast cancers have a higher ALCAM staining intensity compared with tamoxifen sensitive ones. Similar to our results, they showed that knockdown of ALCAM enhanced 4‐hydroxytestosterone induced cell death in tamoxifen resistant breast cancer cell line.^46^ Darvishi et al,^47^ in a further study described that ALCAM blockade resensitized resistant breast cancer cells to tamoxifen. The role of ALCAM in ENZA resistance of PC has not been revealed yet. In the present study, we found that higher pretreatment ALCAM levels are independently associated with shorter OS of ENZA‐treated CRPC patients and further increase of serum ALCAM concentrations at 3 months of treatment was associated with shorter OS. These data suggest ALCAM as a potential biomarker for therapy monitoring in CRPC.

Our study has some potential limitations. First, as no ALCAM expression could be observed in DuCaP and DuCaP‐ENZA cell lines (by LC‐MS/MS and Western‐blot) siRNA mediated ALCAM silencing could only be performed in one cell line pair (LAPC4 and LAPC4‐ENZA). Second, we could only assess one cohort per treatment and could therefore not confirm our results in an independent validation cohort. As our serum analyses have been performed in a retrospective manner, selection bias between different treatment groups (eg, former local therapy, imbalanced numbers regarding therapy lines) may be present. Finally, as we did not assess other potential resistance markers (eg, AR‐V7) we cannot concurrently evaluate the prognostic value of serum ALCAM in this context.

Taken together, serum ALCAM concentration is a potential serum marker of shorter survival in ENZA‐treated patients and ALCAM seems to be functionally involved in ENZA resistance. Our further analyses in serum samples of ABI and DOC‐treated patients revealed serum ALCAM as a predictor of OS in androgen signaling inhibitor‐treated (ABI and ENZA) men but not in DOC chemotherapy‐treated patients. This discriminately effect was even more obvious when ALCAM was combined with PSA (Figures S5,S6 and S7A,B). Moreover, based on subgroup analyses ALCAM levels to be able to identify patients deriving benefit from ENZA&ABI treatment regardless to the treatment line. Therefore, serum ALCAM analysis may discriminate between ENZA/ABI and DOC responsive patients. Our results however, need to be confirmed in larger studies. According to results of our functional analyses, we assume that ALCAM might be mechanistically involved in ENZA resistance and may therefore serve as a potential target for PC therapy. Further analyses are warranted to understand the exact role of ALCAM in ENZA and ABI resistance.

## 5 CONCLUSION

In summary, our analyses revealed, for the first time, that high pretreatment serum ALCAM levels are independently associated with poor survival in ENZA‐ and ABI but not in DOC‐treated CRPC patients. Therefore, ALCAM may help to identify ENZA‐ and ABI‐resistant patients and thereby help to optimize therapeutic decision‐making on type and timing of systemic therapy. However, this analysis has to be validated in larger independent patient cohort. Furthermore, our in vitro functional analyses showed that ALCAM gene silencing in ENZA‐resistant cells leads to enhanced ENZA sensitivity.

## AUTHOR CONTRIBUTIONS

The study reported in the paper has been performed by the authors, unless clearly specified in the text. Anita Csizmarik and Tibor Szarvas were the project administrator and wrote the original manuscript draft. Anita Csizmarik, Péter Nyirády, Boris Hadaschik and Tibor Szarvas concuptalized the study. Anita Csizmarik, Dávid Keresztes, Nikolett Nagy, Tibor Szarvas, József Lázár, Ilona Tornyi, László Takács, Gero Kramer, Sabina Sevcenco, Agnieszka Maj‐Hes. and Zsolt Jurányi conducted the data curation. Anita Csizmarik, Dávid Keresztes, Thilo Bracht, Barbara Sitek, Kathrin Witzke, Ilona Tornyi, József Lázár and László Takács contributed to the methodology. Anita Csizmarik, Dávid Keresztes, Thilo Bracht and Tibor Szarvas created the visualiziations. Dávid Keresztes, Nikolett Nagy, Thilo Bracht, Barbara Sitek, Kathrin Witzke, Martin Puhr, Ilona Tornyi, József Lázár, László Takács, Gero Kramer, Sabina Sevcenco, Agnieszka Maj‐Hes, Zsolt Jurányi, Boris Hadaschik, Péter Nyirády and Tibor Szarvas reviewed and edited the manuscript. Anita Csizmarik, Dávid Keresztes, Nikolett Nagy and Tibor Szarvas performed the formal analysis. Tibor Szarvas and Péter Nyirády supervised the project. Tibor Szarvas acquired funding.

## FUNDING INFORMATION

This study was supported by the National Research, Development and Innovation Office—NKFIH/FK 124431. Tibor Szarvas was supported by a János Bolyai Research Scholarship of the Hungarian Academy of Sciences. Supported by the ÚNKP‐21‐5‐SE‐3 and ÚNKP‐21‐3‐II‐SE‐13 New National Excellence Program of the Ministry for Innovation and Technology from the source of the National Research, Development and Innovation Fund. A part of this study was funded by P.U.R.E. (Protein Research Unit Ruhr within Europe), Ministry of Innovation, Science and Research of North‐Rhine Westphalia, Germany.

## CONFLICT OF INTEREST

Boris Hadaschik reported consulting fees from Bayer, Janssen, Novartis, BMS, AstraZeneca, MSD, Pfizer, Amgen and Astellas. The other authors declare no potential conflicts of interest.

## ETHICS STATEMENT

The study was performed in accordance with the ethical standards of the Helsinki Declaration and was approved by the ethical boards of the hospitals (TUKEB 55/2014, ECS 1986/2017). All patients signed consent to an institutional review board‐approved protocol before sample collection.

## Supporting information


**APPENDIX S1** Supporting Information.

**TABLE S1** Output dataset of LC‐MS/MS based proteome analysis of LAPC4 vs LAPC4‐ENZA cell lines.

**TABLE S2** Output dataset of LC‐MS/MS based proteome analysis of DuCaP vs DuCaP‐ENZA cell lines.

**TABLE S3** Association of baseline AGR2, IDH1 and NDRG1 levels with ENZA‐treated patients' clinicopathological parameters. Significant values are indicated in bold.

## Data Availability

The raw mass spectrometry data generated in this study have been deposited in the PRIDE database with accession number PXD031847 (http://www.ebi.ac.uk/pride). Other data that support the findings of this study are available from the corresponding author upon request.

## References

[1] de Bono JS , Oudard S , Ozguroglu M , et al. Prednisone plus cabazitaxel or mitoxantrone for metastatic castration‐resistant prostate cancer progressing after docetaxel treatment: a randomised open‐label trial. Lancet. 2010;376:1147‐1154. 20888992 10.1016/S0140-6736(10)61389-X

[2] Ryan CJ , Smith MR , Fizazi K , et al. Abiraterone acetate plus prednisone versus placebo plus prednisone in chemotherapy‐naive men with metastatic castration resistant prostate cancer (COU‐AA‐302): final overall survival analysis of a randomised, double‐blind, placebo‐controlled phase 3 study. Lancet Oncol. 2015;16:152‐160. 25601341 10.1016/S1470-2045(14)71205-7

[3] de Bono JS , Logothetis CJ , Molina A , et al. Abiraterone and increased survival in metastatic prostate cancer. N Engl J Med. 2011;364:1995‐2005. 21612468 10.1056/NEJMoa1014618 PMC3471149

[4] Beer TM , Armstrong AJ , Rathkopf DE , et al. Enzalutamide in metastatic prostate cancer before chemotherapy. N Engl J Med. 2014;371:424‐433. 24881730 10.1056/NEJMoa1405095 PMC4418931

[5] Scher HI , Fizazi K , Saad F , et al. Increased survival with enzalutamide in prostate cancer after chemotherapy. N Engl J Med. 2012;367:1187‐1197. 22894553 10.1056/NEJMoa1207506

[6] Kantoff PW , Higano CS , Shore ND , et al. Sipuleucel‐T immunotherapy for castration‐resistant prostate cancer. N Engl J Med. 2010;363:411‐422. 20818862 10.1056/NEJMoa1001294

[7] de Bono J , Mateo J , Fizazi K , et al. Olaparib for metastatic castration‐resistant prostate cancer. N Engl J Med. 2020;382:2091‐2102. 32343890 10.1056/NEJMoa1911440

[8] Abida W , Patnaik A , Campbell D , et al. Rucaparib in men with metastatic castration‐resistant prostate cancer harboring a BRCA1 or BRCA2 gene alteration. J Clin Oncol. 2020;38:3763‐3772. 32795228 10.1200/JCO.20.01035 PMC7655021

[9] Sartor O , Coleman R , Nilsson S , et al. Effect of radium‐223 dichloride on symptomatic skeletal events in patients with castration‐resistant prostate cancer and bone metastases: results from a phase 3, double‐blind, randomised trial. Lancet Oncol. 2014;15:738‐746. 24836273 10.1016/S1470-2045(14)70183-4

[10] Sartor O , de Bono J , Chi KN , et al. Lutetium‐177‐PSMA‐617 for metastatic castration‐resistant prostate cancer. N Engl J Med. 2021;385:1091‐1103. 34161051 10.1056/NEJMoa2107322 PMC8446332

[11] Csizmarik A , Hadaschik B , Kramer G , Nyirady P , Szarvas T . Mechanisms and markers of resistance to androgen signaling inhibitors in patients with metastatic castration‐resistant prostate cancer. Urol Oncol. 2021;39(728):e13‐728.e24. 10.1016/j.urolonc.2021.01.030 33637400

[12] Conteduca V , Wetterskog D , Sharabiani MTA , et al. Androgen receptor gene status in plasma DNA associates with worse outcome on enzalutamide or abiraterone for castration‐resistant prostate cancer: a multi‐institution correlative biomarker study. Ann. Oncol. 2017;28:1508‐1516. 28472366 10.1093/annonc/mdx155 PMC5834043

[13] Antonarakis ES , Lu C , Luber B , et al. Clinical significance of androgen receptor splice variant‐7 mRNA detection in circulating tumor cells of men with metastatic castration‐resistant prostate cancer treated with first‐and second‐line abiraterone and enzalutamide. J Clin Oncol. 2017;35:2149‐2156. 28384066 10.1200/JCO.2016.70.1961 PMC5493048

[14] Conteduca V , Scarpi E , Salvi S , et al. Plasma androgen receptor and serum chromogranin A in advanced prostate cancer. Sci Rep. 2018;8:15442. 30337589 10.1038/s41598-018-33774-4 PMC6194135

[15] Szarvas T , Csizmarik A , Fazekas T , et al. Comprehensive analysis of serum chromogranin A and neuron‐specific enolase levels in localized and castration resistant prostate cancer. BJU Int. 2021;127:44‐55. 32314509 10.1111/bju.15086

[16] Annala M , Vandekerkhove G , Khalaf D , et al. Circulating tumor DNA genomics correlate with resistance to abiraterone and enzalutamide in prostate cancer. Cancer Discov. 2018;8:444‐457. 29367197 10.1158/2159-8290.CD-17-0937

[17] Chen WS , Aggarwal R , Zhang L , et al. Genomic drivers of poor prognosis and enzalutamide resistance in metastatic castration‐resistant prostate cancer. Eur Urol. 2019;76:562‐571. 30928160 10.1016/j.eururo.2019.03.020 PMC6764911

[18] Isaacsson Velho P , Fu W , Wang H , et al. Wnt‐pathway activating mutations are associated with resistance to first‐line abiraterone and enzalutamide in castration‐resistant prostate cancer. Eur Urol. 2020;77:14‐21. 31176623 10.1016/j.eururo.2019.05.032 PMC6893106

[19] Abida W , Cyrta J , Heller G , et al. Genomic correlates of clinical outcome in advanced prostate cancer. Proc Natl Acad Sci U S A. 2019;116:1428‐11436. 10.1073/pnas.1902651116 PMC6561293 31061129

[20] Szarvas T , Csizmarik A , Váradi M , et al. The prognostic value of serum MMP‐7 levels in prostate cancer patients who received docetaxel, abiraterone, or enzalutamide therapy. Urol Oncol. 2020;39(296):e11‐296.e19. 10.1016/j.urolonc.2020.09.005 33046366

[21] Hoefer J , Akbor M , Handle F , et al. Critical role of androgen receptor level in prostate cancer cell resistance to new generation antiandrogen enzalutamide. Oncotarget. 2016;7:59781‐59794. 27486973 10.18632/oncotarget.10926 PMC5312348

[22] Qian S , Xia J , Liu H , Zhang Y , Zhang L , Yu Y . Integrative transcriptome analysis identifies genes and pathways associated with enzalutamide resistance of prostate cancer. Aging Male. 2018;21:231‐237. 29316842 10.1080/13685538.2018.1424129

[23] Scher HI , Halabi S , Tannock I , et al. Design and end points of clinical trials for patients with progressive prostate cancer and castrate levels of testosterone: recommendations of the Prostate Cancer Clinical Trials Working Group. J Clin Oncol. 2008;26:1148‐1159. 18309951 10.1200/JCO.2007.12.4487 PMC4010133

[24] Schneider CA , Rasband WS , Eliceiri KW . NIH image to ImageJ: 25 years of image analysis. Nat Methods. 2012;9:671‐675. 22930834 10.1038/nmeth.2089 PMC5554542

[25] Bartha A , Gyorffy B . TNMplot.com: A web tool for the comparison of gene expression in Normal, tumor and metastatic tissues. Int J Mol Sci. 2021;22:2622. 33807717 10.3390/ijms22052622 PMC7961455

[26] Cerami E , Gao J , Dogrusoz U , et al. The cBio cancer genomics portal: an open platform for exploring multidimensional cancer genomics data. Cancer Discov. 2012;2:401‐404. Erratum in: Cancer Discov. 2012; 2: 960. 22588877 10.1158/2159-8290.CD-12-0095 PMC3956037

[27] Gao J , Aksoy BA , Dogrusoz U , et al. Integrative analysis of complex cancer genomics and clinical profiles using the cBioPortal. Sci Signal. 2013;6:pl1. 23550210 10.1126/scisignal.2004088 PMC4160307

[28] Garri C , Howell S , Tiemann K , et al. Identification, characterization and application of a new peptide against anterior gradient homolog 2 (AGR2). Oncotarget. 2018;9:27363‐27379. 29937991 10.18632/oncotarget.25221 PMC6007958

[29] Tian SB , Tao KX , Hu J , et al. The prognostic value of AGR2 expression in solid tumours: a systematic review and meta‐analysis. Sci Rep. 2017;7:15500. 29138453 10.1038/s41598-017-15757-z PMC5686151

[30] Zhao L , Lee BY , Brown DA , et al. Identification of candidate biomarkers of therapeutic response to docetaxel by proteomic profiling. Cancer Res. 2009;69:7696‐7703. 19773444 10.1158/0008-5472.CAN-08-4901

[31] Tang D , He J , Dai Y , et al. Targeting KDM1B‐dependent miR‐215‐AR‐AGR2‐axis promotes sensitivity to enzalutamide‐resistant prostate cancer. Cancer Gene Ther. 2021;29:543‐557. doi:10.1038/s41417-021-00332-6 33854217

[32] Cancer Genome Atlas Research Network . The molecular taxonomy of primary prostate cancer. Cell. 2015;163:1011‐1025. 26544944 10.1016/j.cell.2015.10.025 PMC4695400

[33] Park KC , Paluncic J , Kovacevic Z , Richardson DR . Pharmacological targeting and the diverse functions of the metastasis suppressor, NDRG1, in cancer. Free Radic Biol Med. 2020;157:154‐175. 31132412 10.1016/j.freeradbiomed.2019.05.020

[34] Zheng L , Dou X , Ma X , Qu W , Tang X . Identification of potential key genes and pathways in enzalutamide‐resistant prostate cancer cell lines: A bioinformatics analysis with data from the gene expression omnibus (GEO) database. Biomed Res Int. 2020;2020:8341097. 32724813 10.1155/2020/8341097 PMC7382728

[35] Darvishi B , Boroumandieh S , Majidzadeh‐A K , Salehi M , Jafari F , Farahmand L . The role of activated leukocyte cell adhesion molecule (ALCAM) in cancer progression, invasion, metastasis and recurrence: A novel cancer stem cell marker and tumor‐specific prognostic marker. Exp Mol Pathol. 2020;115:104443. 32380056 10.1016/j.yexmp.2020.104443

[36] Kahlert C , Weber H , Mogler C , et al. Increased expression of ALCAM/CD166 in pancreatic cancer is an independent prognostic marker for poor survival and early tumour relapse. Br J Cancer. 2009;101:457‐464. 19603023 10.1038/sj.bjc.6605136 PMC2720248

[37] Tomita K , van Bokhoven A , Jansen CF , et al. Activated leukocyte cell adhesion molecule (ALCAM) expression is associated with poor prognosis for bladder cancer patients. UroOncology. 2003;3:121‐129.

[38] Weichert W , Knösel T , Bellach J , Dietel M , Kristiansen G . ALCAM/CD166 is overexpressed in colorectal carcinoma and correlates with shortened patient survival. J Clin Pathol. 2004;57:1160‐1164. 15509676 10.1136/jcp.2004.016238 PMC1770486

[39] King JA , Ofori‐Acquah SF , Stevens T , Al‐Mehdi A‐B , Fodstad O , Jiang WG . Activated leukocyte cell adhesion molecule in breast cancer: prognostic indicator. Breast Cancer Res. 2004;6:R478‐R487. 15318930 10.1186/bcr815 PMC549164

[40] Mezzanzanica D , Fabbi M , Bagnoli M , et al. Subcellular localization of activated leukocyte cell adhesion molecule is a molecular predictor of survival in ovarian carcinoma patients. Clin Cancer Res. 2008;14:1726‐1733. 18347173 10.1158/1078-0432.CCR-07-0428

[41] Kristiansen G , Pilarsky C , Wissmann C , et al. ALCAM/CD166 is up‐regulated in low‐grade prostate cancer and progressively lost in high‐grade lesions. Prostate. 2003;54:34‐43. 12481253 10.1002/pros.10161

[42] Minner S , Kraetzig F , Tachezy M , et al. Low activated leukocyte cell adhesion molecule expression is associated with advanced tumor stage and early prostate‐specific antigen relapse in prostate cancer. Hum Pathol. 2011;42:1946‐1952. 21683980 10.1016/j.humpath.2011.02.017

[43] Kristiansen G , Pilarsky C , Wissmann C , et al. Expression profiling of microdissected matched prostate cancer samples reveals CD166/MEMD and CD24 as new prognostic markers for patient survival. J Pathol. 2005;205:359‐376. 15532095 10.1002/path.1676

[44] Hansen AG , Arnold SA , Jiang M , et al. ALCAM/CD166 is a TGF‐β‐responsive marker and functional regulator of prostate cancer metastasis to bone. Cancer Res. 2014;74:1404‐1415. 24385212 10.1158/0008-5472.CAN-13-1296 PMC4149913

[45] Sanders AJ , Owen S , Morgan LD , et al. Importance of activated leukocyte cell adhesion molecule (ALCAM) in prostate cancer progression and metastatic dissemination. Oncotarget. 2019;10:6362‐6377. 31695844 10.18632/oncotarget.27279 PMC6824871

[46] Chen MJ , Cheng YM , Chen CC , Chen YC , Shen CJ . MiR‐148a and miR‐152 reduce tamoxifen resistance in ERþ breast cancer via downregulating ALCAM. Biochem Biophys Res Commun. 2017;483:840‐846. 28063929 10.1016/j.bbrc.2017.01.012

[47] Darvishi B , Salehi M , Boroumandieh S , et al. Dual in vitro invasion/migration suppressing and tamoxifen response modulating effects of a recombinant anti‐ALCAM scFv on breast cancer cells. Cell Biochem Funct. 2020;38:651‐659. 32196701 10.1002/cbf.3525

[48] Castaneda CA , Castillo M , Enciso JA , et al. Role of undifferentiation markers and androgen receptor expression in triple‐negative breast cancer. Breast J. 2019;25:1316‐1319. 31332870 10.1111/tbj.13464

