# Supplementary material for: Proteome profiling of enzalutamide‐resistant cell lines and serum analysis identified ALCAM as marker of resistance in castration‐resistant prostate cancer
Source: Int J Cancer. 2022 Jun 21;151(8):1405–19. doi: 10.1002/ijc.34159 (PMC9539937; doi:10.1002/ijc.34159)
Supplement: Supplementary file 1 — APPENDIX S1 Supporting Information. [file IJC-151-1405-s001.pdf]

Proteome profiling of enzalutamide-resistant cell lines and serum analysis identified ALCAM as marker of resistance in castration-resistant prostate cancer

Anita Csizmarik, Dávid Keresztes, Nikolett Nagy, Thilo Bracht, Barbara Sitek, Kathrin Witzke, Martin Puhr, Ilona Tornyi, József Lázár, László Takács, Gero Kramer, Sabina Sevcenco, Agnieszka Maj-Hes, Zsolt Jurányi, Boris Hadaschik, Péter Nyirády, Tibor Szarvas

Table of Content:

1. Supplementary Materials and Methods (Pages 2 - 4)
2. Supplementary Tables [S1-S5] (Pages 5 - 6)

    Supplementary Table S1: Output dataset of LC-MS/MS based proteome analysis of LAPC4 vs. LAPC4-ENZA cell lines. Table is available in separate file.

    Supplementary Table S2: Output dataset of LC-MS/MS based proteome analysis of DuCaP vs. DuCaP-ENZA cell lines. Table is available in separate file.

    Supplementary Table S3: Association of baseline AGR2, IDH1 and NDRG1 levels with ENZA-treated patients' clinicopathological parameters. Significant values are indicated in bold. Table is available in separate file.

    Supplementary Table S4: Association of baseline ALCAM levels with clinical parameters in ENZA, ABI and DOC-treated patients. Significant values are indicated in bold. (Page 5)

    Supplementary Table S5: Pearson's and Spearman's correlation of baseline ALCAM levels with clinical parameters in ENZA, ABI and DOC-treated patients. Significant values are indicated in bold. (Page 6)

3. Supplementary Figures [S1 – S9] (Pages 7 - 15)

    Supplementary Figure S1: Flow-chart diagram of the results of LC-MS/MS analysis and biomarker selection. (Page 7)

    Supplementary Figure S2: Gene Ontolgy (GO) analysis (Page 8)

    Supplementary Figure S3: KEGG pathway analysis (Page 9)

    Supplementary Figure S4: Kaplan-Meier overall survival curves show that high ALCAM baseline serum levels are associated with poor OS in ENZA&ABI 1st line treated patients. (Page 10)

Supplementary Figure S5: Kaplan-Meier OS curves in patients with (A) low and high baseline ALCAM levels and (B) ALCAM and PSA combination stratified by the applied therapy (ENZA/ABI and DOC). (Page 11)
Supplementary Figure S6: Kaplan-Meier OS curves in patients with (A) low and high baseline ALCAM levels and (B) ALCAM and PSA combination stratified by the applied therapy in 1st line subgroup (ENZA/ABI and DOC). (Page 12)
Supplementary Figure S7: Kaplan-Meier OS curves in patients with (A) low and high baseline ALCAM levels and (B) ALCAM and PSA combination stratified by the applied therapy in last line subgroup (ENZA/ABI and DOC). (Page 13)
Supplementary Figure S8: mRNA expression of AR and their target genes PSA, c-myc and FKBP5 in LAPC4, LAPC4-ENZA and LAPC4-ENZA siALCAM cells. (Page 14)
Supplementary Figure S9: *In silico* analysis of ALCAM gene expression using TNMplot and cBioPortal databases. (Page 15)

Supplementary Materials and Methods

Detailed description of LC-MS/MS analysis

Sample preparation of cells for Label-free LC-MS/MS

ENZA sensitive (parental) and resistant PC cell lines were harvested at about 80% confluence. We lysed the cell pellets using 0.1% NaDOC in TBS supplemented with protease inhibitors (complete mini, Roche, Penzberg, Germany) and Benzonase (25 U per sample, Merck). Then, we sonicated the samples on ice for 10 min and we added lysis buffer for protein solubilization (30 mM Tris, 7M urea, 2 M thiourea, 0.1% SDS, pH 8.5). We centrifuged the samples at 16100 x g for 10 min. We measured the supernatant concentrations using the Bradford assay (Bio-Rad, Hercules, CA). 30 µg of proteins were loaded to 18% Tris-Glycine-Gels (Anamed Elektrophorese, Rodau, Germany) and allowed to run into the gel (15 min at 100 V). The protein bands were stained with Coomassie and cut from the gels. In-gel trypsin digestion was performed in 10 mM ammonium bicarbonate buffer overnight at 37 °C. The generated peptides were extracted in a vacuum centrifuge and peptides were dissolved in 0.1% TFA. Peptide concentrations were measured via amino acid analysis as described before (Megger et al., Mol Cell Proteomics. 2013 Jul;12(7):2006-20.).

LC-MS/MS Parameters

LC–MS/MS analysis was performed as described previously (Megger et al., Mol Cell Proteomics. 2013 Jul;12(7):2006-20.). Briefly, 300 ng tryptic digested proteins were injected to an Ultimate 3000 RSLCnano HPLC coupled to an Orbitrap Elite instrument (both Thermo Sientific, Bremen, Germany). Peptides were concentrated on a C18 trap column (Acclaim PepMap 100; 100 µm × 2 cm, 5 µm, 100 Å) at a flow rate of 30 µl/min with 0.1% TFA for 7 min. Peptides were transferred to a Nano Viper C18 analytical column (Acclaim PepMap RSLC; 75 µm × 50 cm, 2 µm, 100 Å) and separated with a gradient from 5%–40% solvent B over 98 min at 400 nl/min and 60°C (solvent A: 0.1% FA; solvent B: 0.1% FA, 84% ACN). Full-scan mass spectra were operated in profile mode at a resolution of 60,000 at 400 m/z within a mass range of 350–2000 m/z. MS/MS spectra were acquired at a resolution of 5,400. For MS/MS measurements, the 20 most abundant peptide ions were fragmented by collision-induced dissociation (CID, NCE 35).

Protein Identification and Quantification

We used the Proteome Discoverer v.1.4 (Thermo Fisher Scientific) for protein identification. Spectra were searched against the UniProtKB/Swiss-Prot database (Release 2016_05; 70625 entries) using Mascot v.2.5 (Matrix Science, London, UK). The following search parameters were applied: Homo sapiens taxonomy, precursor ion mass tolerance of 5 ppm and fragment ion mass tolerance of 0.4 Da, dynamic and static modifications methionine (oxidation) and cysteine (carbamidomethyl). The false discovery rate (FDR) was estimated with the Target Decoy PSM Validator and identifications with an FDR > 1% were rejected. We used Progenesis QI v.2.0.5387.52102 (Nonlinear Dynamics, Durham, NC, USA) for label-free quantification. Raw files were aligned to a reference run and a master map of features was applied to all experimental runs to adjust for differences in retention time. Ion charge states of 2+, 3+, and 4+ with a minimum of three isotope peaks were considered. Statistical analysis was done using R and t-tests were calculated using arcsinh-transformed normalized protein abundances. We determined the ratios of mean abundances (RoM) based on non-transformed data. Proteins quantify with minimum two unique peptides and those passing the applied significance thresholds (p-value < 0.05) were considered as significantly differentially abundant.

Real-Time quantitative PCR

Total RNA was isolated using the RNeasy mini kit (Qiagen, Hilden, Germany) and cDNA synthesis was done with a Luna Script RT Super Mix Kit (New England Biolabs, Ipswich, USA). Quantitative RT-PCR was performed on a Bio-Rad CFX system (Bio-Rad Laboratories, Feldkirchen, Germany) using a Luna Universal Probe qPCR Master Mix (New England Biolabs) according to the manufacturer`s protocols. All expression data were normalized the mean of the used housekeeping genes TBP and HPRT1. Custom primers were used at a concentration of 800 nmol/L each and FAM-TAMRA/BHQ1 labeled probes at 150 nmol/L. Primer sequences for AR, KLK3, TBP and HPRT1 are as follows: AR: Fwd: 5'-AGGATGCTCTACTTCGCCCC-3'; Rev: 5'-ACTGGCTGTACATCCGGGAC-3'; probe: 5'-FAM-TGGTTTTCAATGAGTACCGCATGCACA-TAMRA-3', KLK3: Fwd: 5'-GTCTGCGGCGGTGTTCTG-3'; Rev: 5'-TGCCGACCCAGCAAGATC-3'; probe: 5'-FAM-CACAGCTGCCCACTGCATCAGGA-TAMRA-3', TBP: Fwd: 5'-

CACGAACCACGGCACTGATT-3', Rev: 5'-TTTTCTTGCTGCCAGTCTGGAC-3', probe: 5`-FAM-TCTTCACTCTTGGCTCCTGTGCACA-TAMRA-3`, HPRT1: Fwd: 5`-GCTTTCCTTGGTCAGGCAGTA-3`, Rev: 5`-GTCTGGCTTATATCCAACACTTCGT-3`, probe: 5`-FAM-TCAAGGTCGCAAGCTTGCTGGTGAAAAGGA-TAMRA- 3`. TaqMan gene-expression assays (Thermo Fisher Scientific) were used for c-myc (Hs00153408_m1) and FKBP5 (Hs01561006_m1).

Supplemetary Tables

Supplementary Table S4: Association of baseline ALCAM levels with clinical parameters in ENZA, ABI and DOC-treated patients. Significant values are indicated in bold.

RPE - radical prostatectomy

RAD - radiation

	ENZA			ABI			DOC		
	n<br>72	ALCAM serum cc. (ng/ml) median (range)	P	n<br>101	ALCAM serum cc. (ng/ml) median (range)	P	n<br>100	ALCAM serum cc. (ng/ml) median (range)	P
Primary RPE									
no	43	119.5 (82.5 - 400.0)	0.209	53	118.1 (45.2 - 400.0)	0.801	82	114.2 (51.9 - 400.0)	0.333
yes	29	146.3 (0 - 400.0)		48	122.3 (49.6 - 389.2)		18	118.9 (47.2 - 242.8)	
Primary RAD									
no	55	140.2 (0 - 400.0)	0.101	80	122.7 (49.6 - 400.0)	0.569	88	126.7 (47.2 - 400.0)	0.110
yes	17	117.1 (84.8 - 256.1)		21	117.1 (45.2 - 389.2)		12	93.8 (64.9 - 219.8)	
Pain									
no	46	122.4 (0 - 400.0)	0.075	50	115.9 (45.2 - 389.2)	**0.013**	-	-	-
yes	19	153.9 (83.5 - 400.0)		36	135.8 (56.8 - 400.0)		-	-	
unknown	7			15			-		
PSA decline									
yes	60	121.1 (0 - 400.0)	0.133	87	119.9 (45.2 - 400.0)	0.284	15	107.4 (60.5 - 343.8)	0.597
no	12	148.9 (90.6 - 203.1)		14	126.3 (67.3 - 233.8)		71	117.8 (47.2 - 400.0)	
unknown	0			0			14		
PSA decline									
> 50%	47	118.3 (0 - 400.0)	0.083	65	122.2 (45.2 - 400.0)	0.972	45	107.8 (57.6 - 400.0)	0.551
< 50%	25	146.3 (68.3 - 400.0)		36	118.8 (55.1 - 286.0)		41	126.5 (47.2 - 400.0)	
unknown	0			0			14		
PSA decline									
> 90%	26	117.1 (82.3 - 400.0)	0.253	33	122.2 (52.9 -361.0)	0.834	26	107.2 (57.6 - 400.0)	0.683
< 90%	46	143.3 (0 - 400.0)		68	120.2 (45.2 - 400.0)		60	137.4 (47.2 -400.0)	
unknown	0			0			14		

Supplementary Table S5: Pearson's and Spearman's correlation of baseline ALCAM levels with clinical parameters in ENZA, ABI and DOC-treated patients. Significant values are indicated in bold.

LDH - Lactate dehydrogenase

CRP - C reactive protein

AP - Alkaline phosphatase

Pearson's	ENZA			ABI			DOC		
	ALCAM			ALCAM			ALCAM		
	n	corr coef	p	n	corr coef	p	n	corr coef	p
Age	72	0.106	0.376	101	0.746	**0.033**	100	0.113	0.263
PSA	72	0.631	**<0.001**	101	0.428	**<0.001**	100	0.371	**<0.001**
LDH	69	0.591	0.066	100	0.430	**<0.001**	27	0.936	**0.016**
AP	69	0.508	**<0.001**	99	0.192	0.056	31	0.235	0.202
CRP	68	0.217	0.075	100	0.024	0.811	31	0.121	0.515
Hemoglobin	70	0.297	**0.013**	99	0.152	0.133	30	0.191	0.311

Spearman's	ENZA			ABI			DOC		
	ALCAM			ALCAM			ALCAM		
	n	corr coef	p	n	corr coef	p	n	corr coef	p
Age	72	0.159	0.183	101	0.054	0.588	100	0.108	0.286
PSA	72	0.570	**<0.001**	101	0.366	**<0.001**	100	0.348	**<0.001**
LDH	69	0.470	**<0.001**	100	0.303	**0.002**	27	0.176	0.379
AP	69	0.305	**0.011**	99	0.339	**0.001**	31	0.277	0.132
CRP	68	0.298	**0.014**	100	0.179	0.075	31	0.201	0.278
Hemoglobin	70	0.315	**0.008**	99	0.207	**0.040**	30	0.196	0.299

Supplemetary Figures

Supplementary Figure S1: Flow-chart diagram of the results of LC-MS/MS analysis and biomarker selection. For details of filtering methods please see materials and methods section of the manuscript.

↑ - upregulated proteins

↓ - downregulated proteins

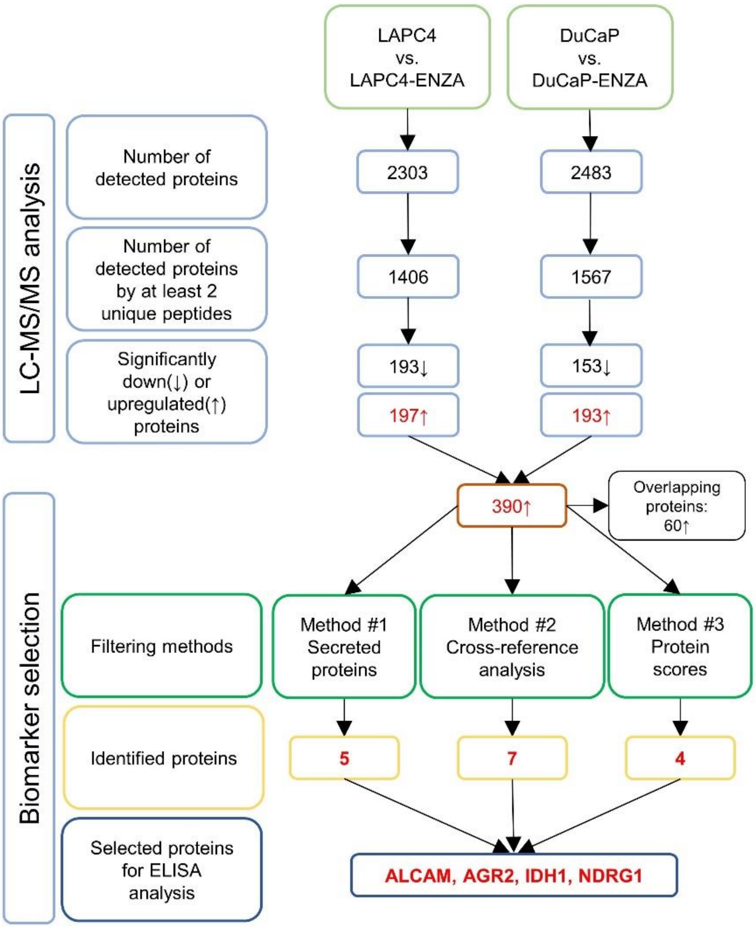

Supplementary Figure S2: Gene Ontolgy (GO) analysis. Proteins significantly up- and downregulated in ENZA-resistant cells were analyzed using the STRING database (v.11.5). Enriched ontologies (Biological Process) were filtered (observed gene count ≥ 25, false discovery rate ≤ 0.0001, strength ≥ 0.25, background gene count ≤ 3000) and manually reviewed to remove redundant and inapplicable terms. Proteins are displayed with point size corresponding to the observed gene count and the color corresponding to the significance of enrichment.

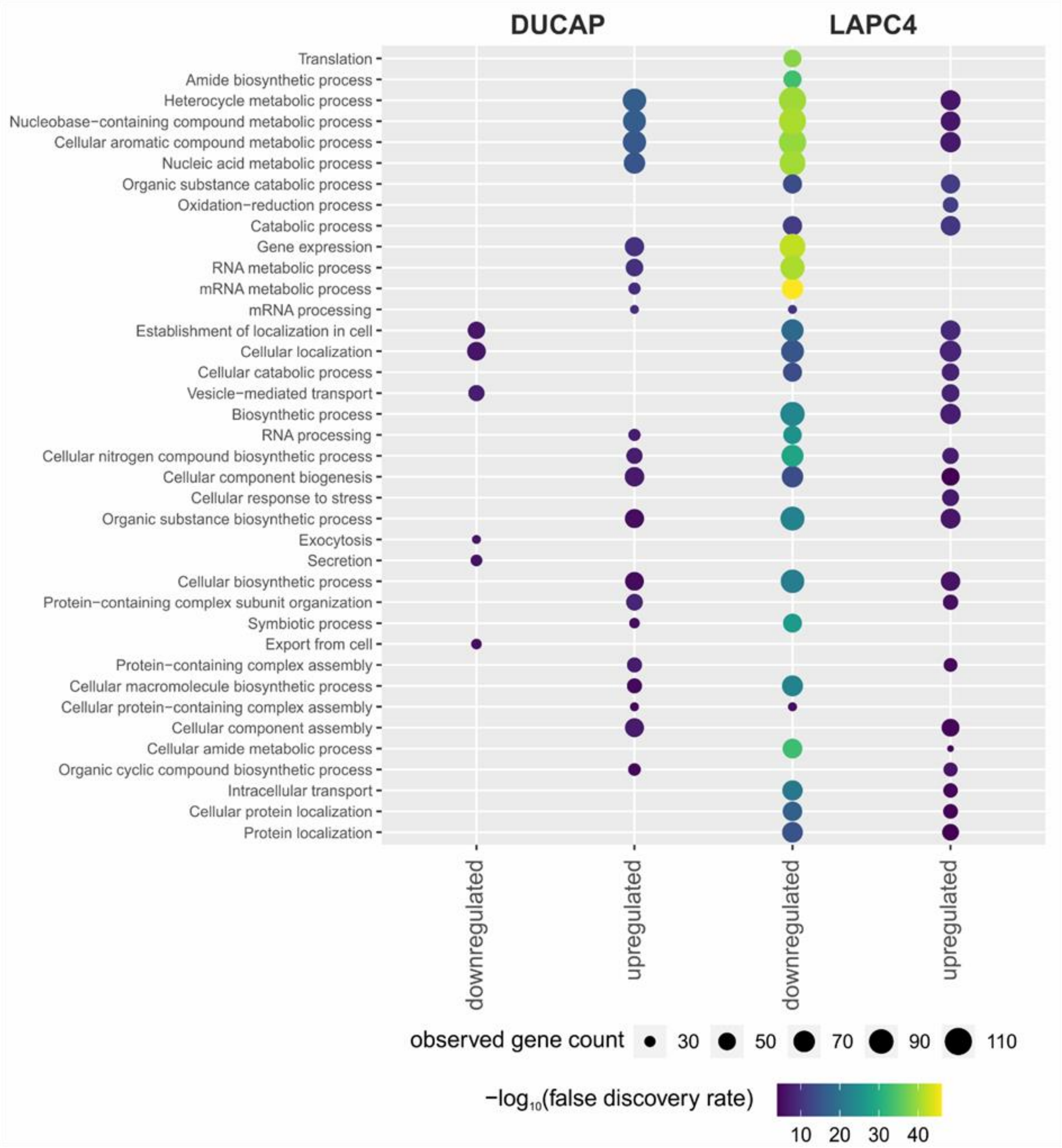

Supplementary Figure S3: KEGG pathway analysis. Proteins significantly down- and upregulated in ENZA-resistant cells, respectively, were analyzed for DuCaP and LAPC4 cell lines using the STRING database (v.11.5). The enriched KEGG pathways were manually reviewed to remove inapplicable terms. Proteins displayed with point size corresponding to the observed gene count and the color corresponding to the significance of enrichment.

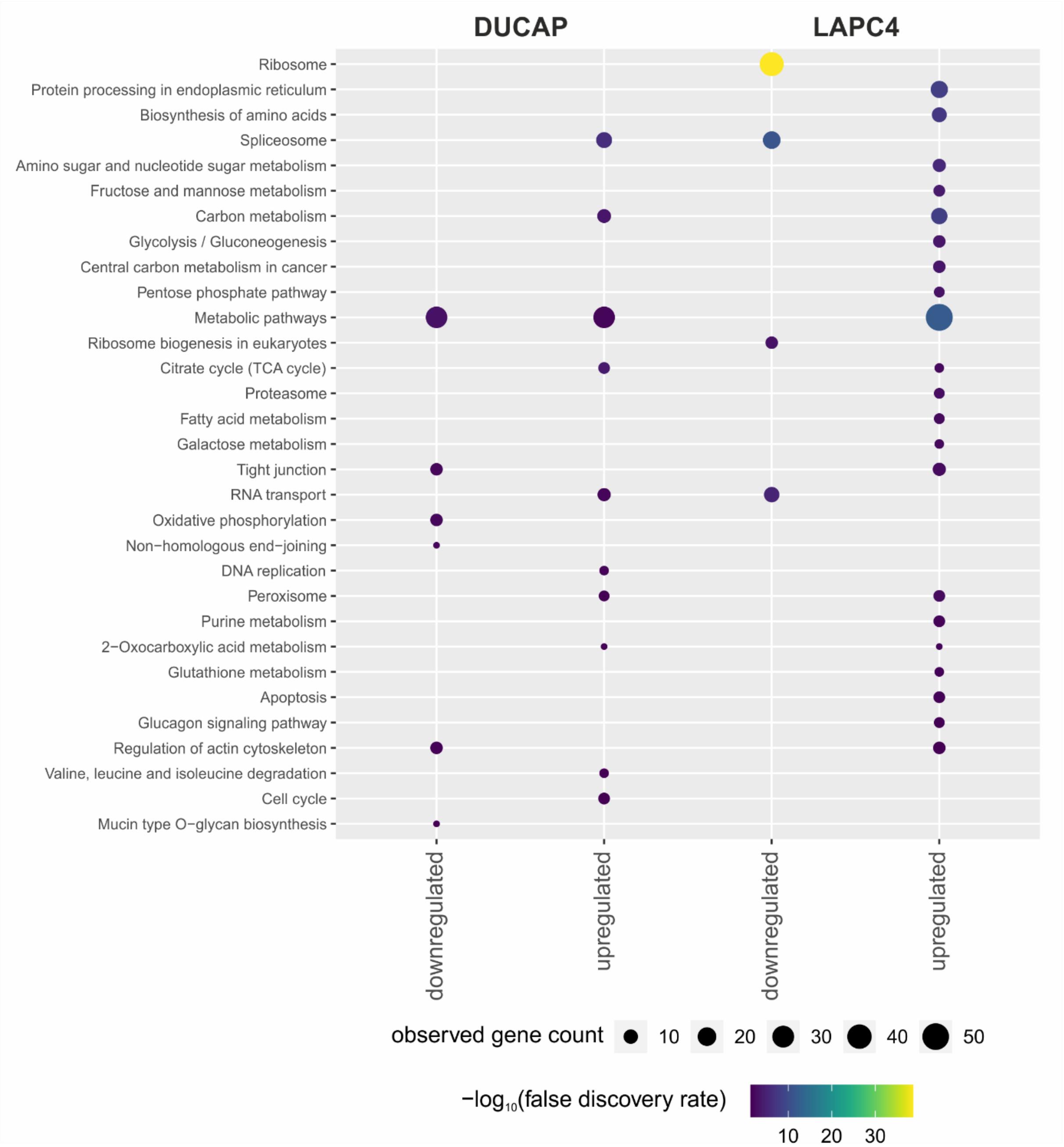

Supplementary Figure S4: Kaplan-Meier overall survival curves show that high ALCAM baseline serum levels are associated with poor OS in ENZA&ABI 1st line treated patients

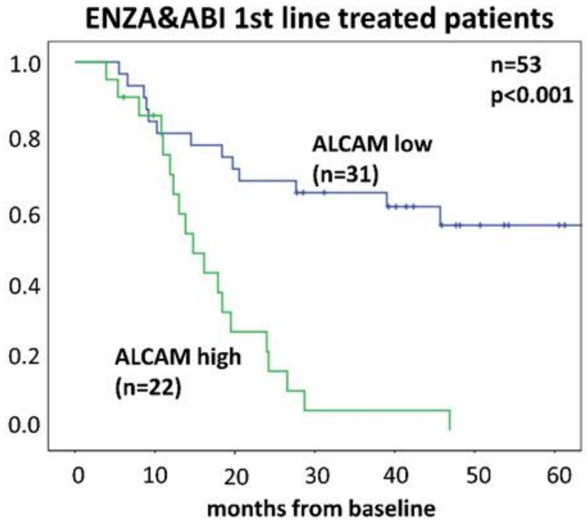

Supplementary Figure S5: Kaplan-Meier OS curves in patients with (A) low and high baseline ALCAM levels and (B) ALCAM and PSA combination stratified by the applied therapy (ENZA/ABI and DOC).

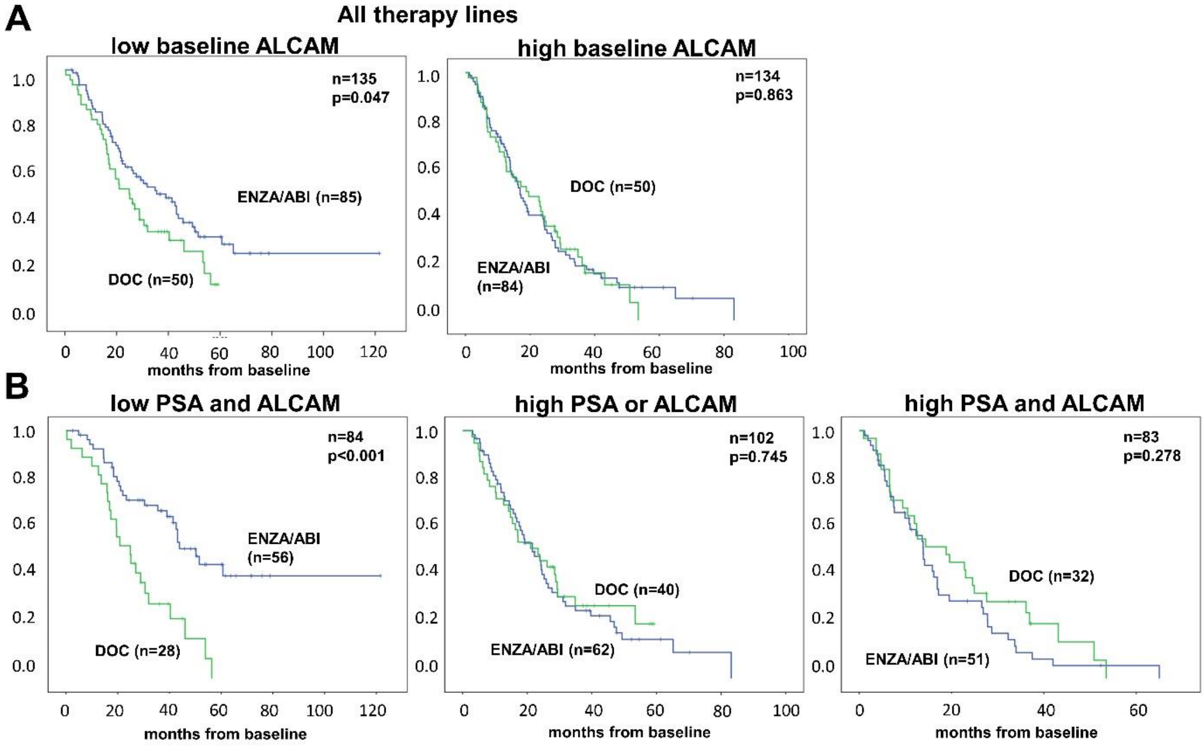

Supplementary Figure S6: Kaplan-Meier OS curves in patients with (A) low and high baseline ALCAM levels and (B) ALCAM and PSA combination stratified by the applied therapy in 1st line subgroup (ENZA/ABI and DOC).

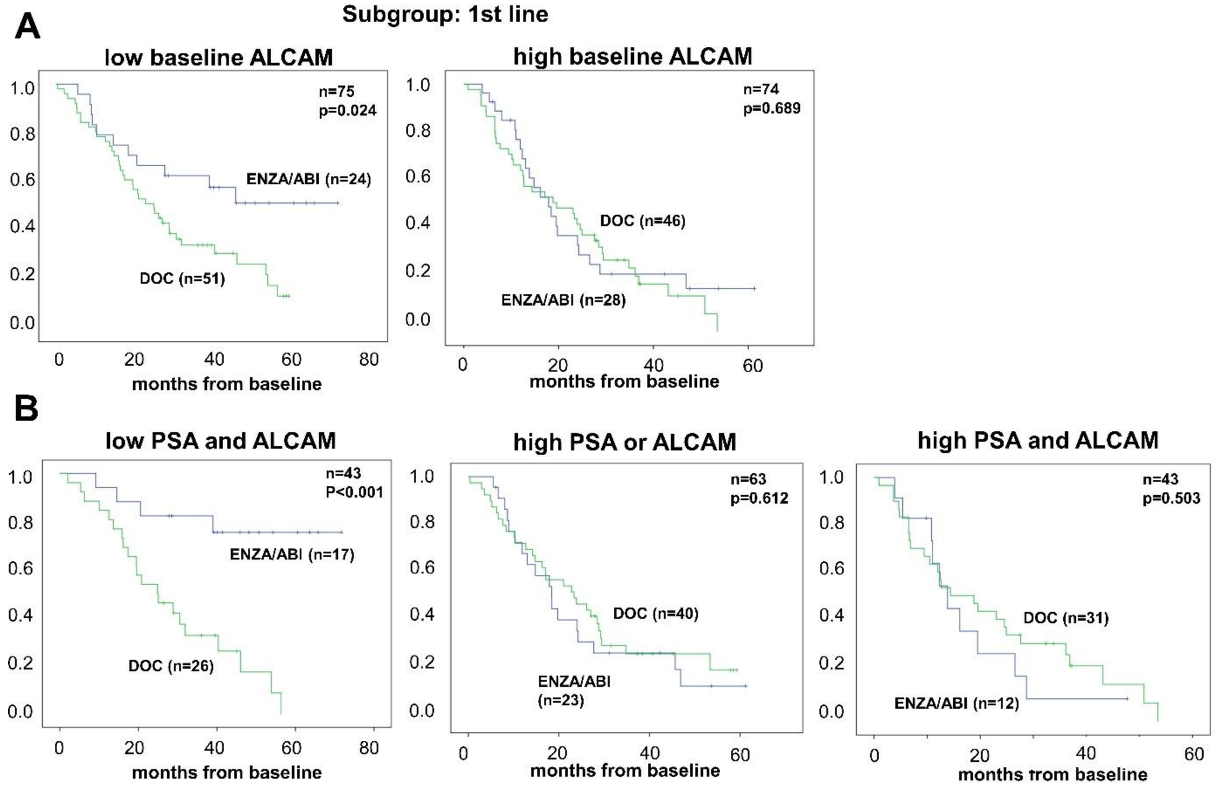

Supplementary Figure S7: Kaplan-Meier OS curves in patients with (A) low and high baseline ALCAM levels and (B) ALCAM and PSA combination stratified by the applied therapy in last line subgroup (ENZA/ABI and DOC).

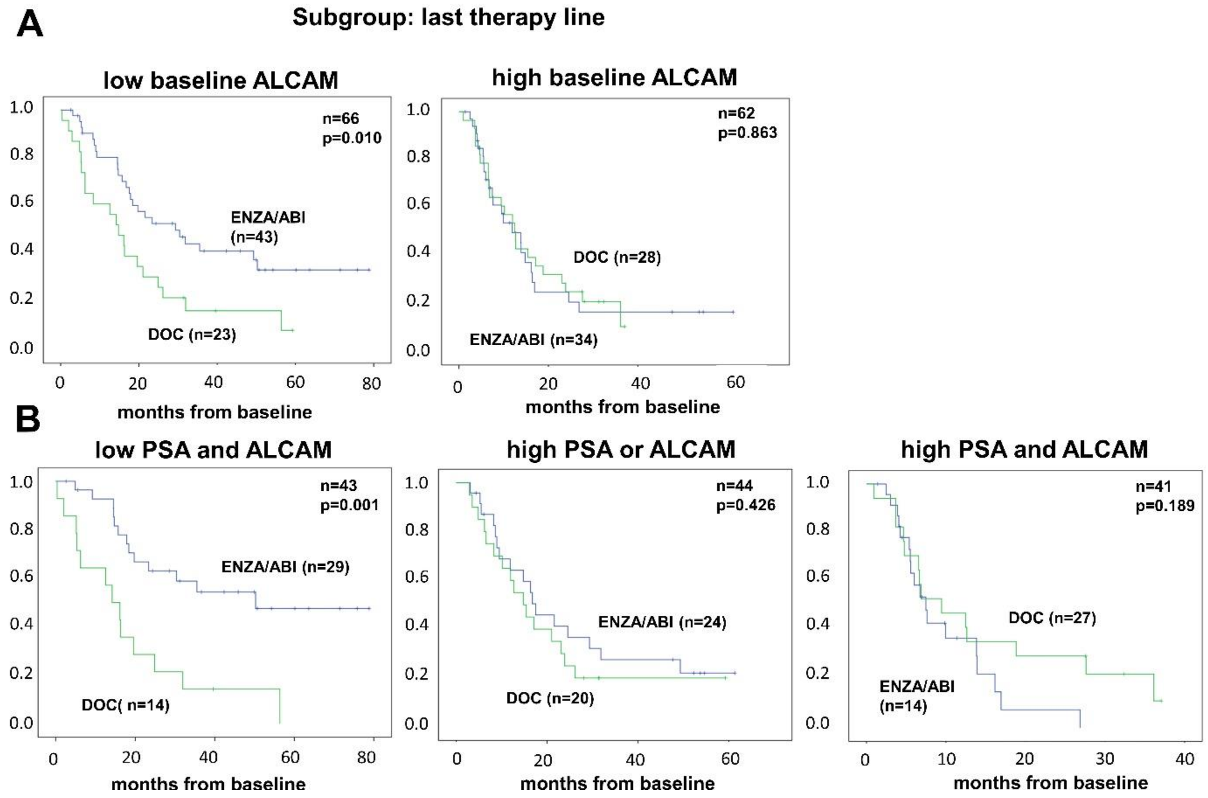

Supplementary Figure S8: mRNA expression of AR and their target genes PSA, c-myc and FKBP5 in LAPC4, LAPC4-ENZA and LAPC4-ENZA siALCAM cells. Data were presented as mean from three independent experiments. *p < 0.05

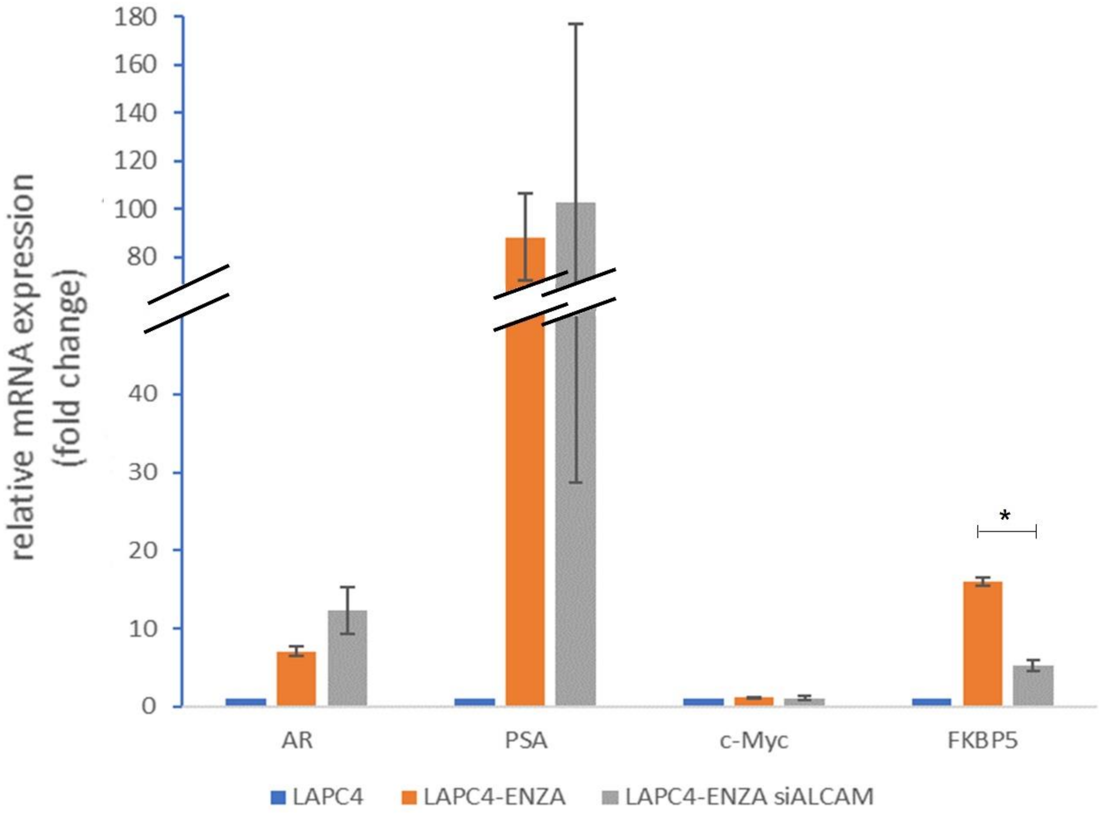

Supplementary Figure S9: *In silico* analysis of ALCAM gene expression using the TNMplot and cBioPortal databases. (A) Box-plot presentation of ALCAM expression analysis in various normal and corresponding tumor tissues in the TNMplot database. Significant differences are marked with red fonts *P < 0.05. (B) Box-plot presentation of ALCAM gene expression in PC from the TNMplot database. ALCAM showed higher expression in metastatic and tumor PC samples compared to normal tissues. (C) ALCAM gene expression showed no significant correlation with overall survival (p=0.270) based on TCGA data (D) Correlation between ALCAM mRNA expression with molecular and clinical data from TCGA data using cBioPortal database. (E) ALCAM showed significantly higher expression in PC adenocarcinoma compared to small cell and NEPC (neuroendocrine prostate cancer) variants. (F) ALCAM expressions are significantly associated with high AR gene expression score. (G) Higher ALCAM gene expressions are associated with lower NEPC gene expression score.

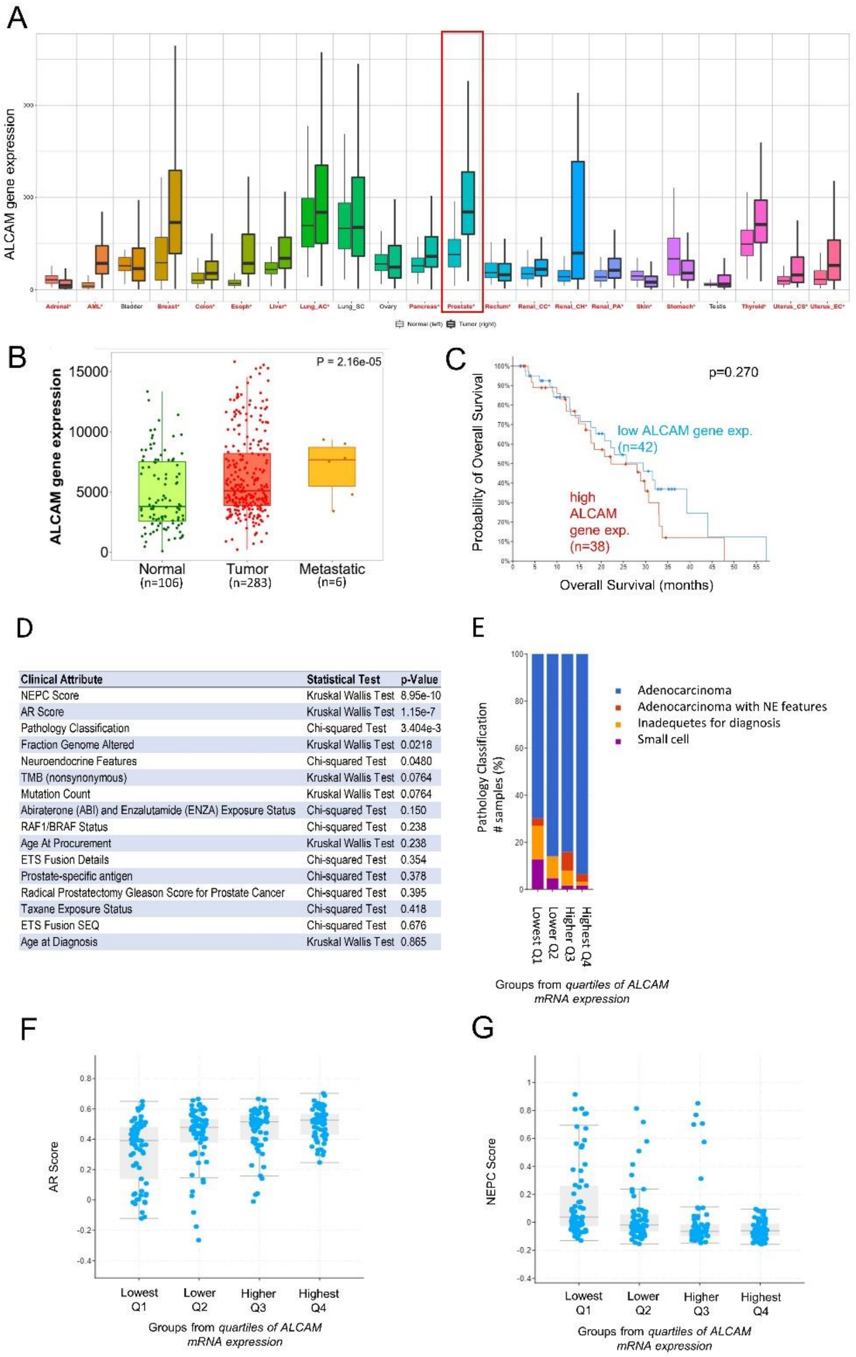

Clinical Attribute	Statistical Test	p-Value
NEPC Score	Kruskal Wallis Test	8.95e-10
AR Score	Kruskal Wallis Test	1.15e-7
Pathology Classification	Chi-squared Test	3.404e-3
Fraction Genome Altered	Kruskal Wallis Test	0.0218
Neuroendocrine Features	Chi-squared Test	0.0480
TMB (nonsynonymous)	Kruskal Wallis Test	0.0764
Mutation Count	Kruskal Wallis Test	0.0764
Abiraterone (ABI) and Enzalutamide (ENZA) Exposure Status	Chi-squared Test	0.150
RAF1/BRAF Status	Chi-squared Test	0.238
Age At Procurement	Kruskal Wallis Test	0.238
ETS Fusion Details	Chi-squared Test	0.354
Prostate-specific antigen	Chi-squared Test	0.378
Radical Prostatectomy Gleason Score for Prostate Cancer	Chi-squared Test	0.395
Taxane Exposure Status	Chi-squared Test	0.418
ETS Fusion SEQ	Chi-squared Test	0.676
Age at Diagnosis	Kruskal Wallis Test	0.865
